# Infection-induced 5′-half molecules of tRNA^HisGUG^ activate Toll-like receptor 7

**DOI:** 10.1371/journal.pbio.3000982

**Published:** 2020-12-17

**Authors:** Kamlesh Pawar, Megumi Shigematsu, Soroush Sharbati, Yohei Kirino

**Affiliations:** 1 Computational Medicine Center, Sidney Kimmel Medical College, Thomas Jefferson University, Philadelphia, Pennsylvania, United States of America; 2 Institute of Veterinary Biochemistry, Department of Veterinary Medicine, Freie Universität Berlin, Berlin, Germany; Universiteit Gent, BELGIUM

## Abstract

Toll-like receptors (TLRs) play a crucial role in the innate immune response. Although endosomal TLR7 recognizes single-stranded RNAs, their endogenous RNA ligands have not been fully explored. Here, we report 5′-tRNA half molecules as abundant activators of TLR7. Mycobacterial infection and accompanying surface TLR activation up-regulate the expression of 5′-tRNA half molecules in human monocyte-derived macrophages (HMDMs). The abundant accumulation of 5′-tRNA halves also occur in HMDM-secreted extracellular vehicles (EVs); the abundance of EV-5′-tRNA^HisGUG^ half molecules is >200-fold higher than that of the most abundant EV-microRNA (miRNA). Sequence identification of the 5′-tRNA halves using cP-RNA-seq revealed abundant and selective packaging of specific 5′-tRNA half species into EVs. The EV-5′-tRNA^HisGUG^ half was experimentally demonstrated to be delivered into endosomes in recipient cells and to activate endosomal TLR7. Up-regulation of the 5′-tRNA half molecules was also observed in the plasma of patients infected with *Mycobacterium tuberculosis*. These results unveil a novel tRNA-engaged pathway in the innate immune response and assign the role of “immune activators” to 5′-tRNA half molecules.

## Abstract 2

Although Toll-like receptors (TLRs) play a crucial role in the innate immune response, their endogenous ligands have not been fully explored. This study identifies tRNA half-molecules as abundant TLR ligands which are upregulated upon infection by mycobacteria and activate TLR7.

## Introduction

There are many pathogenic microbes that induce a wide range of symptoms and diseases, including *Mycobacterium tuberculosis* (Mtb), one of the greatest threats to humans, causing more than 1.2 million deaths annually [1]. When a host is infected with pathogenic microbes, it has 2 essential arms of defense to eliminate them: the innate immune system and the adaptive immune system [2]. In the innate immune system, Toll-like receptors (TLRs) and other pathogen recognition receptors detect pathogen-associated molecular patterns (PAMPs) and initiate protective responses [3,4]. Among the 10 TLRs characterized in humans, TLR1, TLR2, TLR4, TLR5, TLR6, and TLR10 localize to the cell surface (surface TLRs), while TLR3, TLR7, TLR8, and TLR9 localize to intracellular compartments such as endosomes (endosomal TLRs). When TLRs recognize PAMPs, they recruit adaptor proteins, such as MyD88 and TRIF, to initiate signal transduction pathways that culminate in the activation of transcription factors such as NF-κB and AP-1, leading to the production of cytokines and chemokines for host defense [5,6].

Endosomal TLRs are known to sense nucleic acids, which act as ligands [7,8]. Of the endosomal TLRs, TLR7 and TLR8 recognize single-stranded RNAs (ssRNAs), whereas TLR3 and TLR9 recognize dsRNAs and ssDNAs, respectively. TLR7 and TLR8 are primarily expressed in immune cells such as monocytes/macrophages, dendritic cells, neutrophils, and B cells, and their recognition of pathogen-derived ssRNAs (e.g., viral and bacterial ssRNAs) recruits MyD88, activates NF-κB-mediated transcription, and induces the production of interferons and cytokines [9]. Besides pathogen-derived ssRNAs, TLR7 and TLR8 also sense host ssRNAs, such as microRNAs (miRNAs). miRNAs can be incorporated into extracellular vehicles (EVs), and those EV-miRNAs can reach and function as agonist of endosomal TLR7 and TLR8 in recipient cells [10–12]. The activation of TLR7 and TLR8 by miRNAs is involved not only in the immune response [13,14]but also in tumor growth and metastasis [15–17] and in neuronal damage and apoptosis [10,18]. Considering that EV contains many other RNA species (e.g., messenger RNAs [mRNAs], transfer tRNAs [tRNAs], small nucleolar RNAs [snoRNAs], Y-RNAs, vault RNAs, and long noncoding RNAs [lncRNAs]) [19,20], it is not surprising that those EV-RNAs are also deliverable to endosomal TLRs and function as their ligands, though this possibility remains unexplored.

Although tRNAs are best known as essential adapter molecules of translational machinery, recent studies have established their role as a source of short noncoding RNAs (ncRNAs) [21–24]. In many organisms, specific tRNA-derived ncRNAs are expressed as functional molecules and are involved in various biological processes beyond translation. tRNA-derived ncRNAs can be classified into 2 groups: tRNA halves and shorter tRNA-derived fragments (tRFs). Among them, 5′-tRNA halves, which comprise the region from the 5′-end to the anticodon loop of tRNAs, are one of the most abundant classes. In mammalian cells, they are generated from angiogenin (ANG)-mediated anticodon cleavage of tRNAs [25,26] and have been shown to regulate translation, promote stress response, promote cell proliferation, and be associated with cancers, neurodegenerative diseases, and metabolic disorders [21–24,27–29]. 5′-tRNA halves further function as direct precursors of Piwi-interacting RNAs (piRNAs) in germ cells [30].

Despite their demonstrated functionality, information regarding the expression profiles of 5′-tRNA halves and their regulation remains elusive, in part because 5′-tRNA halves are not captured by standard RNA sequencing (RNA-seq). As a result of ANG-catalyzed biogenesis, 5′-tRNA halves contain a 2′,3′-cyclic phosphate (cP) at their 3′-end [28,31]. These cP-containing RNAs (cP-RNAs) are not ligated to a 3′-adapter during cDNA amplification, and thus they are not amplified in standard RNA-seq procedures. This limitation remains cP-RNAs, including 5′-tRNA halves, to form uncharacterized components in the transcriptomes [31]. To resolve this issue, we developed “cP-RNA-seq” [28,32], which is able to specifically sequence cP-RNAs and identify a comprehensive expression repertoire of 5′-tRNA halves and other cP-RNA species in human and *Bombyx* cultured cells and mouse tissues [28,30,33,34].

Although the expression of tRNA halves is regulated by various biological factors, such as stresses and sex hormones [25,26,28], how bacterial infection regulates their expression is not fully understood. Here, we report the expressional regulation and functional involvement of 5′-tRNA half molecules in the infection-induced innate immune response. Infection of *Mycobacterium bovis* BCG (BCG) and surface TLR activation induced the expression of 5′-tRNA halves in human monocyte-derived macrophages (HMDMs). cP-RNA-seq-based identification of the induced 5′-tRNA halves in HMDMs and their secreted EVs revealed selective and abundant packaging of 5′-tRNA halves into EVs. We further experimentally demonstrated the delivery of the EV-5′-tRNA halves into endosomes of recipient cells and strong TLR7 activation by 5′-tRNA halves. Induction of the expression and secretion of 5′-tRNA halves was further confirmed in the plasma of Mtb-infected patients, verifying that the observed phenomena occur not only in cell culture systems but also in actual pathological situations. Our study unveils a novel tRNA-engaged pathway in the innate immune response and newly assigned the role of immune activators to 5′-tRNA halves.

## Results

### BCG infection and surface TLR activation induce the expression of 5′-tRNA halves in HMDMs

**Fig 1:**
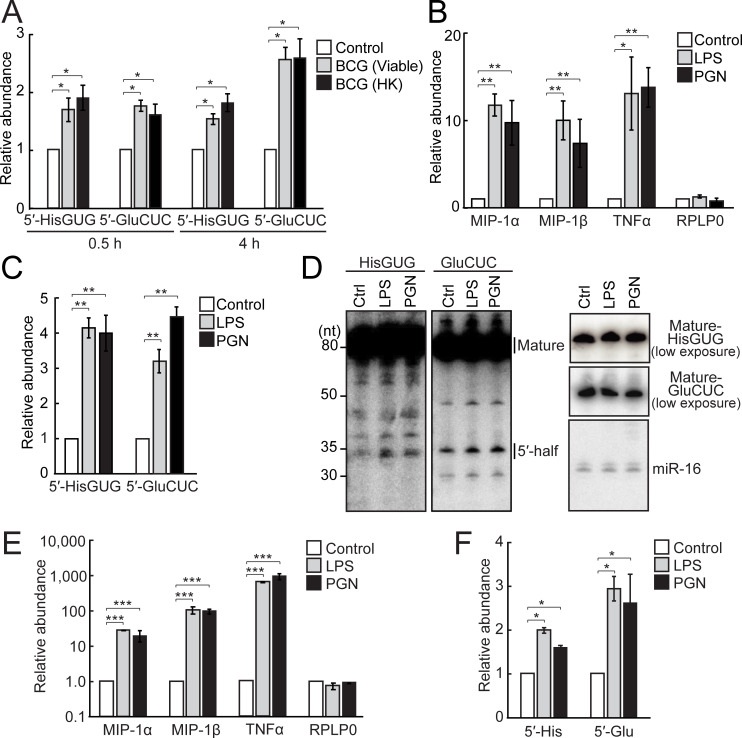
Up-regulation of the expression of 5′-tRNA halves by BCG infection and surface TLR activation. **(A)** Total RNAs from HMDMs infected with viable or HK BCG for 0.5 or 4 h were subjected to TaqMan RT-qPCR for 5′-tRNA^HisGUG^ half (5′-HisGUG) and 5′-tRNA^GluCUC^ half (5′-GluCUC). Noninfected HMDMs served as a control. The quantified 5′-tRNA half levels were normalized to U6 snRNA levels. Averages of 3 experiments with SD values are shown (*P < 0.05, **P < 0.01, and ***P < 0.001; 2-tailed *t* test). **(B, C)** Total RNAs from HMDMs treated with LPS or PGN for 12 h were subjected to RT-qPCR for the indicated mRNAs (B) and to TaqMan RT-qPCR for the 5′-tRNA halves (C). HMDMs without treatment served as a control. The quantified 5′-tRNA half levels were normalized to the levels of U6 snRNA and GAPDH mRNA, respectively. **(D)** Total RNAs from HMDMs treated with LPS or PGN were subjected to northern blot for the 5′-tRNA halves and their corresponding mature tRNAs. miR-16 was analyzed as a control. **(E, F)** Total RNAs from PHMDMs treated with LPS or PGN were subjected to RT-qPCR for the indicated mRNAs (E) and to TaqMan RT-qPCR for the 5′-tRNA halves (F). PHMDMs without treatment served as a control. BCG, *Mycobacterium bovis* BCG; GAPDH, glyceraldehyde 3-phosphate dehydrogenase; HK, heat-killed; HMDM, human monocyte-derived macrophage; LPS, lipopolysaccharide; mRNA, messenger RNA; PGN, peptidoglycan; PHMDM, primary human monocyte-derived macrophage; RT-qPCR, quantitative reverse transcription PCR; SD, standard deviation; TLR, Toll-like receptor; tRNA, transfer tRNA.

HMDMs express both surface and endosomal TLRs and have been used to study TLR pathways [35,36], while BCG has been used as a model bacterium for tuberculosis infection [37]. In the present study, THP-1-derived HMDMs were infected with viable or heat-killed (HK) BCG, and two 5′-tRNA halves (5′-tRNA^HisGUG^ half and 5′-tRNA^GluCUC^ half; previously abundantly detected in human breast cancer cells [28]) were quantified by tRNA half-specific TaqMan quantitative reverse transcription PCR (RT-qPCR) [28,30], in which a 3′-adapter was ligated to the 5′-tRNA half, and then the ligation products were quantified using a TaqMan probe targeting boundary of the adapter and the tRNA half. As shown in **Fig 1A**, BCG infection enhanced the expression of both of the 5′-tRNA halves. The induction of 5′-tRNA half expression was independent of the viability of BCG (**Fig 1A**), suggesting that the induction could result from the pathway of surface TLRs, which recognize BCG PAMPs, or from the process of endocytosis. To examine the involvement of surface TLRs in 5′-tRNA half expression, we stimulated TLR4 and TLR2 by treating HMDMs with lipopolysaccharide (LPS) or peptidoglycan (PGN), respectively [38,39]. Successful stimulations of the TLRs were confirmed by up-regulation of tumor necrosis factor α (TNFα) and the macrophage inflammatory factors, MIP-1α and MIP-1β (**Fig 1B**). Upon stimulation of the surface TLRs, the expression of 5′-tRNA halves was observed to be up-regulated by TaqMan RT-qPCR (**Fig 1C**) and northern blot (**Fig 1D**). Notably, the expression levels of corresponding mature tRNAs were unchanged by surface TLR stimulation (**Fig 1D**). As described in previous studies [26,28,30,40], the production of 5′-tRNA halves did not influence the levels of mature tRNAs which are steadily maintained by an unknown mechanism. We further analyzed primary human monocyte-derived macrophages (PHMDMs) differentiated from CD14+ monocytes. As in the case of HMDMs, treatment of PHMDMs with LPS or PGN caused surface TLR activation (**Fig 1E**) and up-regulation of 5′-tRNA half expression (**Fig 1F**), confirming the surface TLR-induced expression of 5′-tRNA halves in the primary cells of the human body.

### Surface TLR-activated NF-κB up-regulates the expression of ANG mRNA

In mammalian cells, ANG cleaves the anticodon loops of tRNAs to produce tRNA halves [25,26,28]. To confirm the involvement of ANG in the tRNA half production in LPS-treated HMDMs, we performed siRNA-mediated knockdown (KD) of ANG expression, which reduced the ANG mRNA levels to around 35% (**S1A Fig**). The ANG KD decreased the expression of 5′-tRNA halves (**S1B Fig**), suggesting that tRNA halves are generated by ANG-mediated cleavage of tRNAs in LPS-treated HMDMs. Because the expression levels of ANG mRNA were up-regulated upon LPS or PGN treatment in HMDMs (**S1C Fig**) and PHMDMs (**S1D Fig**), we reasoned that the transcription factors downstream of surface TLR signal transduction pathways, such as NF-κB, could induce the expression of ANG mRNA. Indeed, direct binding of NF-κB to the region upstream of the *ANG* gene was suggested by chromatin immunoprecipitation and sequencing (ChIP-seq) data for NF-κB in lymphoblastoid B cells [41] (**S1E Fig**). The potential involvement of NF-κB in ANG mRNA expression was examined by treating HMDMs with JSH-23, an inhibitor of NF-κB [42,43], which reduced the immune response as expected (**S1F Fig**). ANG mRNA levels were unchanged when HMDMs were treated with NF-κB inhibitor and LPS (**S1G Fig**), suggesting that NF-κB-mediated transcription up-regulates ANG mRNA, which would increase the levels of ANG protein, possibly leading to enhanced tRNA cleavage for induction of tRNA half expression by surface TLR activation.

### 5′-tRNA halves are massively accumulated in EVs secreted from HMDMs

**Fig 2:**
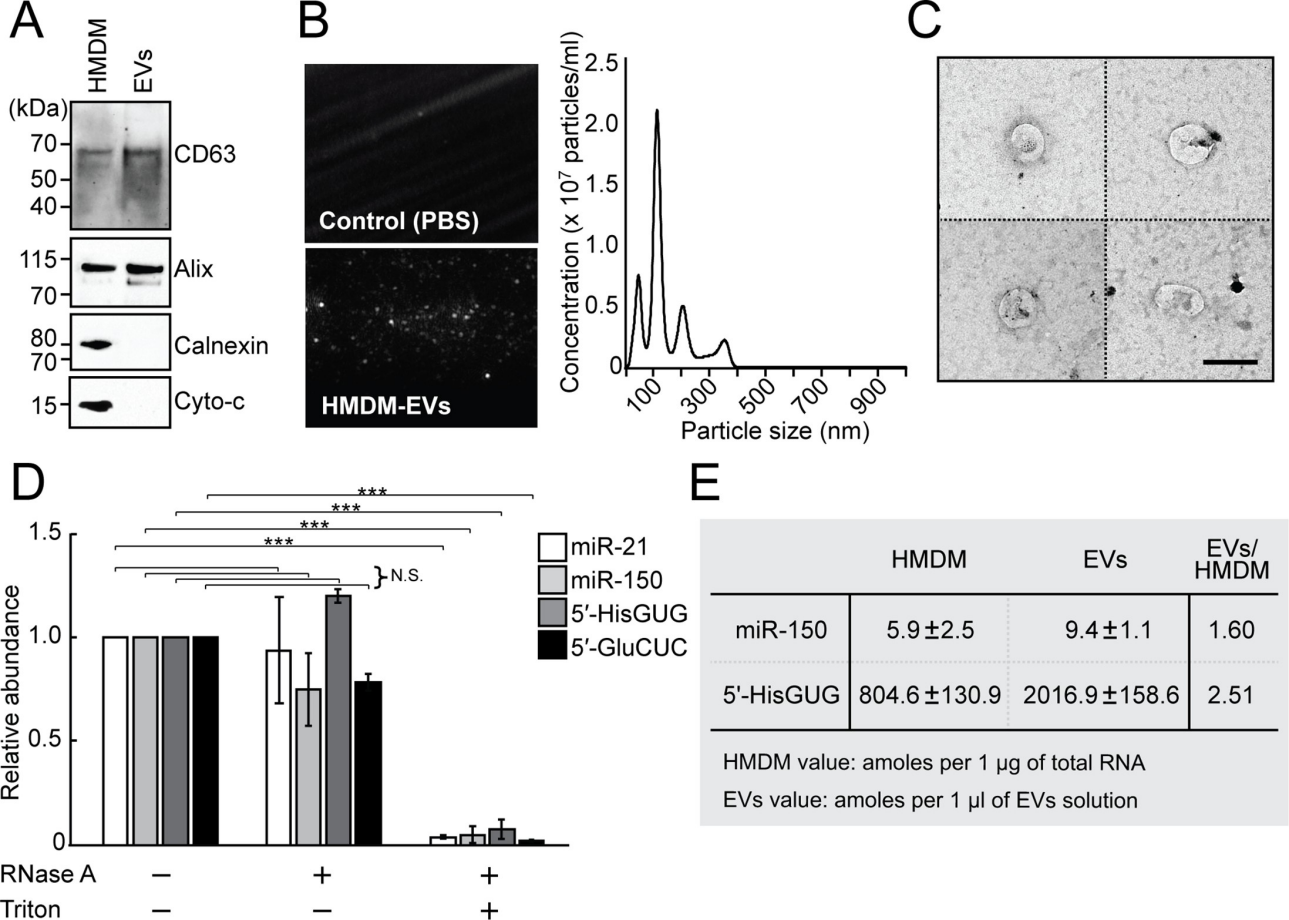
Abundant accumulation of tRNA halves in HMDM-secreted EVs. **(A)** Lysates from HMDMs and their secreted EVs were subjected to western blots for the indicated EV- or non-EV proteins. **(B)** Isolated EVs (HMDM-EVs) were analyzed by NTA. Particle images [left; Control (PBS): negative control] and size distribution profile (right) are shown. Representative raw video files from the NTA analyses are available in S1 and S2 Movies. **(C)** Transmission electron microscopic evaluation for the isolated EVs showed small vesicles with the expected size of EVs. Four representative EV images are shown. Scale bar, 100 nm. **(D)** Isolated EVs were treated with RNase A and/or Triton X-100 and then subjected to stem-loop RT-qPCR and TaqMan RT-qPCR for quantification of each of the 2 indicated miRNAs and 5′-tRNA halves, respectively. Averages of 3 experiments with SD values are shown (***P < 0.001; N.S., nonsignificant, based on 2-tailed *t* test). **(E)** Expression of the miR-150 and 5′-tRNA^HisGUG^ half in HMDMs and their EVs was quantified by stem-loop/TaqMan RT-qPCRs, and their abundance was estimated based on the standard curves shown in **S3 Fig**. Averages of 3 experiments with SD values are shown. Cyto-c, cytochrome-c; EV, extracellular vehicle; HMDM, human monocyte-derived macrophage; miRNA, microRNA; NTA, nanoparticle tracking analysis; RT-qPCR, quantitative reverse transcription PCR; SD, standard deviation; tRNA, transfer tRNA.

To explore whether EVs secreted from HMDMs are carriers of tRNA halves, we isolated the EVs from the culture medium of LPS-treated HMDMs by an ultracentrifugation-based method. Western blots for the isolated EVs confirmed the presence of CD63 and Alix, proteins known for EV accumulation [44,45], and the absence of calnexin and cytochrome c, which are non-EV proteins [46,47] (**Fig 2A**). Nanoparticle tracking analysis (NTA) showed the abundant presence of EVs from 80 to 120 nm at a high concentration (approximately 2.0 × 10^7^ particles/ml EV solution) (**Fig 2B, S1 and S2 Movies**). The isolated EVs were further observed by transmission electron microscopy (**Fig 2C**), the results of which collectively confirmed the successful isolation of HMDM EVs. The isolated EVs were subjected to TaqMan RT-qPCR for two 5′-tRNA halves, 5′-tRNA^HisGUG^ half, and 5′-tRNA^GluCUC^ half, as well as to stem-loop RT-qPCR for 2 miRNAs, miR-21 and miR-150, which are known to abundantly accumulate in HMDM EVs [44]. We obtained clear amplification signals from all of the 4 examined RNAs. While the EVs treated with RNase alone yielded similar amplification signals to untreated EVs, the EVs treated with both RNase and detergent yielded drastically reduced amplification signals (**Fig 2D**), confirming that the detected 5′-tRNA halves and miRNAs were present inside the isolated EVs and were not captured as non-EV contaminants. We further explored the absolute amounts of the 5′-tRNA^HisGUG^ half and miR-150 in LPS-treated HMDMs and their EVs. The calculation of the amounts was based on the standard curve from synthetic RNAs, which showed excellent linearity between input amounts and amplification signals (**S2 and S3 Figs**). The determined abundances of the 2 RNAs per μg of total HMDM RNA or per μl of EV fraction are shown in **Fig 2E**. Although miR-150 was reported as the most abundant miRNA species expressed in HMDMs and their EVs [44], the abundance of the 5′-tRNA^HisGUG^ half was much more pronounced than that of miR-150: 136-fold and 215-fold higher in HMDMs and EVs, respectively.

### 5′-tRNA halves are produced from specific tRNA species in HMDMs and are selectively packaged into EVs

**Fig 3:**
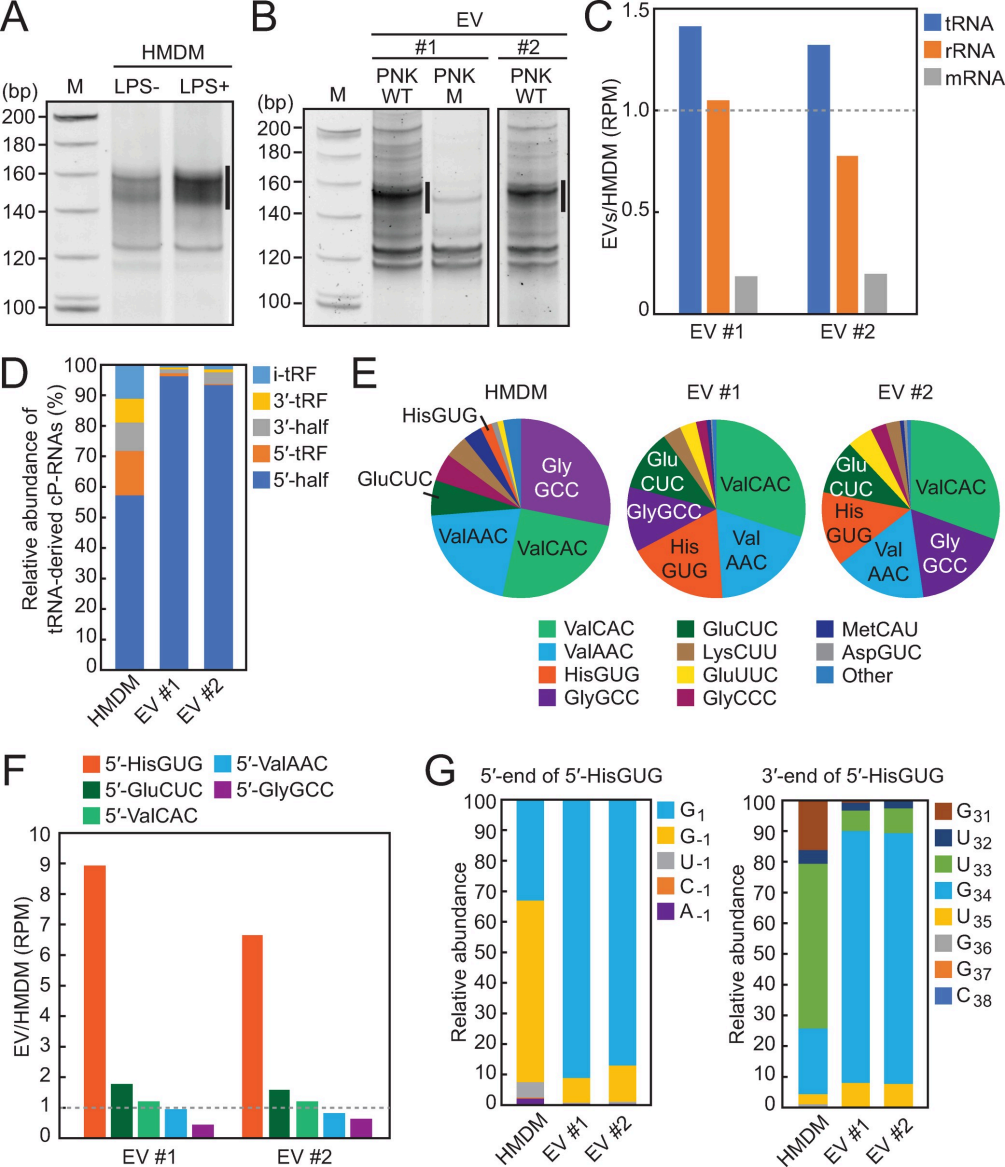
Identification of 5′-tRNA halves expressed in HMDMs and their EVs by cP-RNA-seq. **(A)** Gel-purified 20–45-nt RNAs from LPS-treated HMDMs (untreated HMDMs: control) were subjected to cP-RNA-seq, which amplified 140–160-bp cDNA products (5′-adapter, 55 bp; 3′-adapter, 63 bp; and thereby estimated inserted sequences, 22–42 bp). The cDNAs in the region highlighted by a line were purified and subjected to Illumina sequencing. **(B)** HMDM EV-RNAs (#1 and #2: biological replicates) were treated with WT T4 PNK (PNK WT) or its mutant (PNK M) lacking 3′-dephosphorylation activity and then subjected to Illumina cDNA amplification. Amplification of 140–160-bp cDNA products was dependent on PNK WT treatment. **(C)** Ratio of HMDM library versus EV library for RPM of tRNA-derived RNA reads (tRNA), ribosomal RNA-derived RNA reads (rRNA), and mRNA-derived RNA reads (mRNA). **(D)** Proportion of tRNA-derived cP-RNAs classified into the indicated subgroups of tRNA-derived ncRNAs. 5′- and 3′-tRFs are derived from 5′- and 3′-parts of tRNAs, respectively, while i-tRFs are derived from wholly internal parts of tRNAs [24]. **(E)** Proportion of the 5′-tRNA half-reads derived from respective cyto tRNA species. **(F)** Ratio of HMDM library versus EV library for RPM of the indicated 5′-tRNA half species. **(G)** Proportion of 5′-terminal (left) and 3′-terminal (right) nucleotides of the 5′-tRNA^HisGUG^ halves. cP, 2′,3′-cyclic phosphate; EV, extracellular vehicle; HMDM, human monocyte-derived macrophage; LPS, lipopolysaccharide; mRNA, messenger RNA; nt, nucleotides; RNA-seq, RNA sequencing; RPM, reads per million; T4 PNK, T4 polynucleotide kinase; tRF, tRNA-derived fragment; tRNA, transfer tRNA; WT, wild-type.

Given the abundant accumulation of 5′-tRNA halves in HMDMs and their EVs, we next identified the expression profiles of the 5′-tRNA halves. Although short RNA-seq was previously performed for HMDMs and their EVs [48,49], standard RNA-seq cannot accurately capture 5′-tRNA halves because they possess a cP at their 3′-end that hinders adapter ligation [28]. Instead, we employed “cP-RNA-seq,” which can selectively amplify and sequence cP-RNAs, namely 5′-tRNA halves [28,32]. The cP-RNA-seq procedure was first applied to gel-purified short RNA fractions of HMDMs, which successfully amplified approximately 140- to 160-bp bands (considering adapters’ lengths, inserted RNA sequences were estimated to be approximately 22 to 42 nucleotides [nt] in length) (**Fig 3A**). Consistent with the up-regulation of HMDM tRNA half expression by LPS treatment (**Fig 1**), cP-RNA-seq amplified more abundant cDNAs from the LPS-treated HMDMs than from the untreated cells (**Fig 3A**). In contrast, we failed to amplify clear cDNA bands from the RNAs of HMDM EVs by cP-RNA-seq, possibly due to the limited amounts of EV-RNAs present. The cP-RNA-seq procedure includes a periodate oxidation step, which might be harsh enough to damage whole RNAs if the initial RNA amounts are limited. Therefore, for EV-RNAs, we decided to capture all short RNA species containing not only a cP but also a phosphate (P) or a hydroxyl group (OH) at the 3′-end. For this, EV-RNAs were first treated with T4 polynucleotide kinase (T4 PNK), which can remove cP and P from the 3′-end of RNAs, and then were subjected to the short RNA-seq procedure. This yielded abundant approximately 140- to 160-bp cDNA bands (**Fig 3B**), similar to the bands obtained from cP-RNA-seq of HMDMs (**Fig 3A**). Interestingly, RNAs treated with a mutant T4 PNK, which lacks 3′-dephosphorylation activity [50], yielded only faint cDNA bands, suggesting that the majority of short RNA species in EVs contain a 3′-terminal cP or P and RNAs containing a 3′-OH end, such as miRNAs, are the minor species in EVs; this is consistent with the experimental results shown in **Fig 2E**.

Illumina sequencing of the gel-purified approximately 140- to 160-bp cDNAs from HMDMs and their EVs yielded approximately 35 to 44 million raw reads, of which >82% to 95% were extracted as reads with a length of 25 to 50 nt (**S1 Table**). tRNA-mapped reads were enriched in EV libraries (**Fig 3C**); among them, the 5′-tRNA halves were the most major species, as expected (**Fig 3D**). While 5′-tRNA halves comprised approximately 57% of tRNA-derived reads in HMDMs, they accounted for over 93% of tRNA-derived reads in EVs, suggesting that 5′-tRNA halves could be selectively packaged into EVs to a greater extent than other tRNA-derived RNAs. Considering that the human genome encodes 55 cytoplasmic (cyto) tRNA isoacceptors with different anticodon sequences [51], the identified 5′-tRNA halves were derived from a rather focused subset of tRNAs, such as cyto tRNA^ValCAC^, tRNA^ValAAC^, tRNA^GlyGCC^, tRNA^HisGUG^, and tRNA^GluCUC^, which are in aggregates the sources of 88% to 90% of the identified 5′-tRNA halves in EVs (**Fig 3E**). Among the 5 major 5′-tRNA halves, the relative abundance of the 5′-tRNA^HisGUG^ half in EVs was considerably greater than that in HMDMs, while the other four 5′-tRNA halves were similarly abundant in both libraries (**Fig 3E and 3F**), implying preferential incorporation of the 5′-tRNA^HisGUG^ half into EVs. tRNA^HisGUG^ contains an additional nucleotide at nucleotide position (np; according to the nucleotide numbering system of tRNAs [52]) –1 of its 5′-end. Our recent analyses of BT-474 human breast cancer cells showed that the majority (approximately 60%) of cyto tRNA^HisGUG^ contains G_–1_, but a significant proportion contains U_–1_ or lacks the –1 nucleotide (contains G_1_ as a 5′-terminal nucleotide) [53]. As shown in **Fig 3G**, while the 5′-tRNA^HisGUG^ half containing G_–1_ was the major species in HMDMs, the majority of the 5′-tRNA^HisGUG^ halves in EVs lacked the –1 nucleotide (G_1_). Similarly, while the major 3′-terminal nucleotide was U_33_ for HMDM 5′-tRNA^HisGUG^ halves, the majority of the EV-5′-tRNA^HisGUG^ halves contained G_34_ as the 3′-end. The 5′-tRNA^HisGUG^ half from G_1_ to G_34_ comprised approximately 80% of EV-5′-tRNA^HisGUG^ halves but only 5% of HMDM 5′-tRNA^HisGUG^ halves. Inconsistency of the identified species between HMDMs and EVs was also observed in some other major 5′-tRNA half species (**S4 Fig**), implying that the efficiency of EV loading may not be equal for all 5′-tRNA halves and specific species could be preferentially packaged into EVs.

### EV-5′-tRNA halves are delivered into endosomes in recipient HMDMs

**Fig 4:**
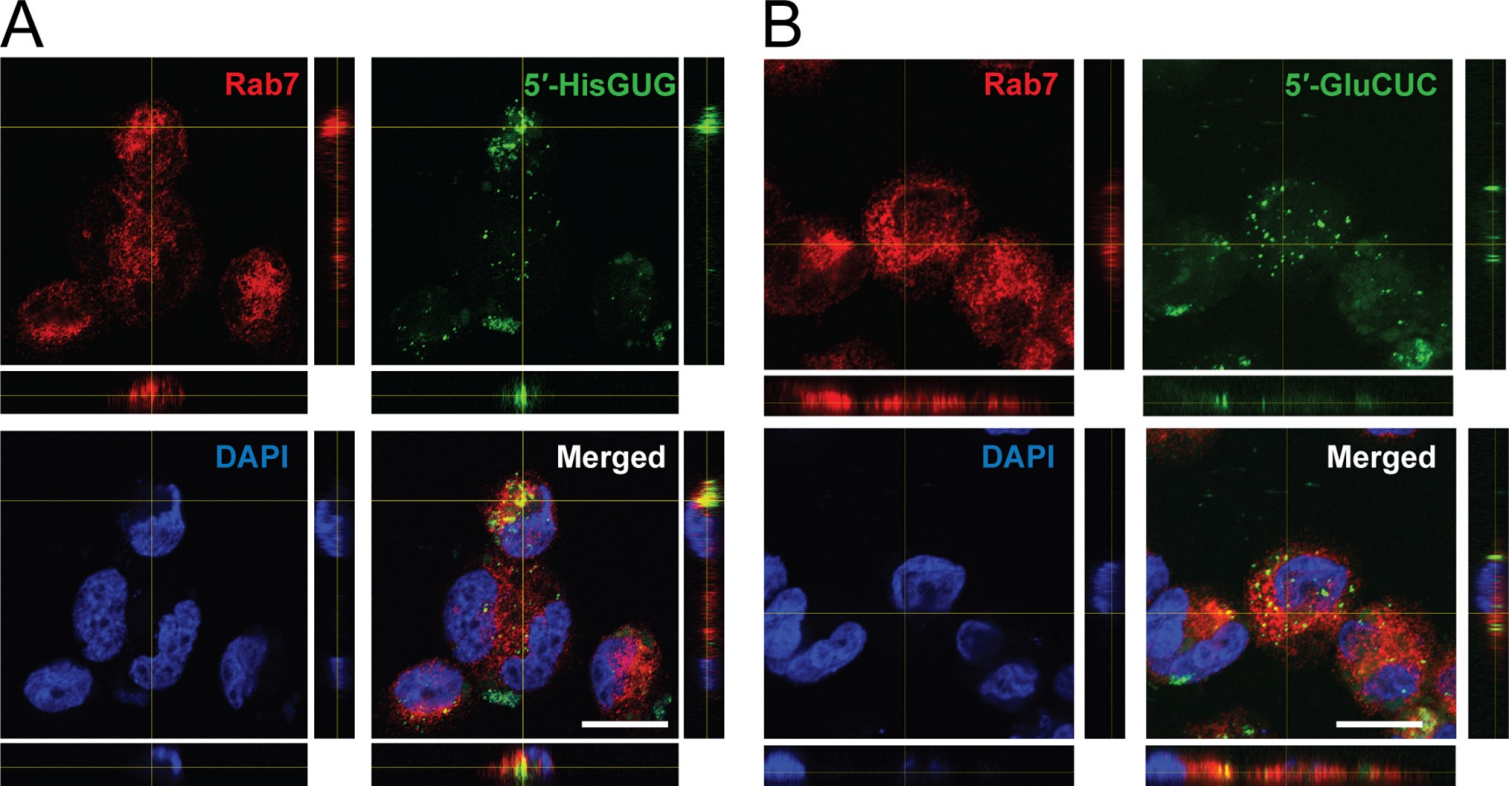
Delivery of EV-5′-tRNA halves into endosomes in recipient cells. EVs produced from host HMDMs containing the labeled 5′-tRNA^HisGUG^ half **(A)** or 5′-tRNA^GluCUC^ half **(B)** were isolated and applied to recipient HMDMs. Delivery of the labeled, EV-5′-tRNA half into endosomes is observed in green. Immunofluorescence staining of Rab7 is shown in red, and DNA was counterstained with DAPI in blue. Clear co-localization of the labeled 5′-tRNA halves and Rab7 is observed in merged panels. Scale bar, 100 μm. EV, extracellular vehicle; HMDM, human monocyte-derived macrophage; tRNA, transfer tRNA.

Because EV-miRNAs have been shown to be ligands for endosomal TLRs [10,11], we examined whether the abundantly identified EV-tRNA halves are delivered into endosomes in recipient cells. We chemically tagged synthetic 5′-tRNA^HisGUG^ half or 5′-tRNA^GluCUC^ half with fluorescein-5-thiosemicarbazide (FTSC) [54] and transfected it into HMDMs, as shown in **S5A and S5B Fig**. We then isolated the EVs containing the labeled 5′-tRNA halves from the transfected cells and subsequently applied them to recipient HMDMs. As a result, we observed the incorporation of the labeled EV-5′-tRNA halves into recipient cells. Clear overlap between the signals of the 5′-tRNA halves and Rab7 (**Fig 4A and 4B**), an endosome marker [55], and TLR7 (**S5C and S5D Fig**) confirmed the delivery of EV-tRNA halves into the endosomes of the recipient HMDMs. These results experimentally proved that tRNA halves in HMDMs are packaged into EVs and secreted outside of the cells, which are then delivered into the endosomes of recipient cells.

### 5′-tRNA^HisGUG^ half activates endosomal TLR7

**Fig 5:**
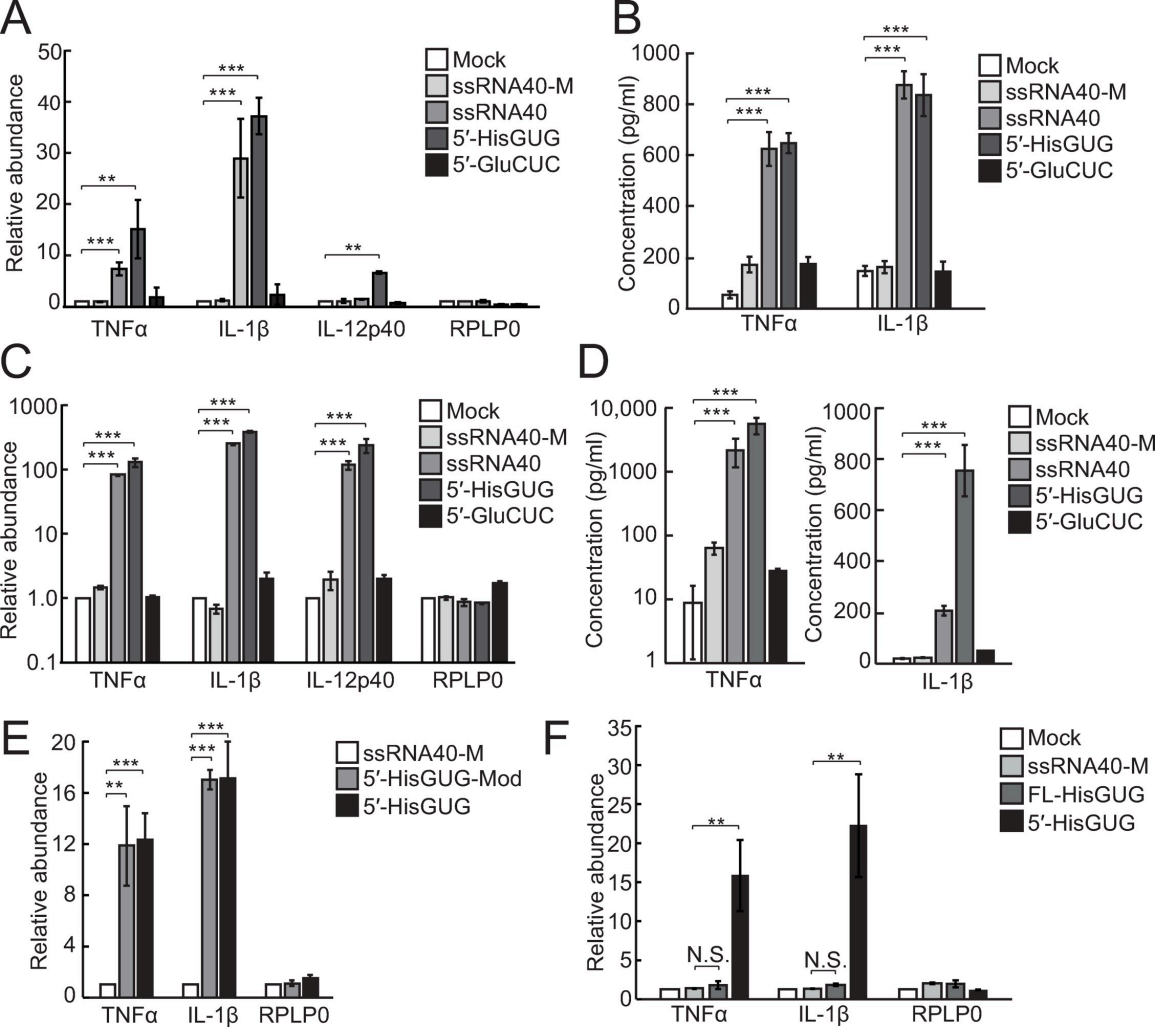
Activation of endosomal TLR by DOTAP-fused 5′-tRNA^HisGUG^ half. **(A)** Using DOTAP, the synthetic 5′-tRNA halves, ssRNA40 (positive control), and its mutant (ssRNA40-M; negative control) were transfected into HMDMs. Total RNAs from the cells were subjected to RT-qPCR for the indicated mRNAs. Averages of 3 experiments with SD values are shown (**P < 0.01 and ***P < 0.001; 2-tailed *t* test). **(B)** After RNA transfection into HMDMs using DOTAP, culture medium was subjected to ELISA for quantification of TNFα and IL-1β. **(C)** The experiments in (A) were performed in PHMDMs. **(D)** The experiments in (B) were performed in PHMDMs. **(E)** The experiments in (A) were performed using 5′-tRNA^HisGUG^ half with modifications (5′-HisGUG-Mod). **(F)** The experiments in (A) were performed using full-length tRNA^HisGUG^ (FL-HisGUG). DOTAP, 1,2-dioleoyloxy-3-trimethylammonium-propane; HMDM, human monocyte-derived macrophage; IL, interleukin; mRNA, messenger RNA; PHMDM, primary human monocyte-derived macrophage; RT-qPCR, quantitative reverse transcription PCR; SD, standard deviation; TLR, Toll-like receptor; TNFα, tumor necrosis factor α; tRNA, transfer tRNA.

Given the abundant accumulation and endosome-targeted delivery of 5′-tRNA halves in HMDM EVs, we further assessed the activity of the 5′-tRNA halves in stimulating ssRNA-sensing endosomal TLRs (i.e., TLR7 and TLR8). As described in earlier studies [11,56], HMDMs were primed with interferon γ and then transfected with 5′-tRNA^HisGUG^ half or 5′-tRNA^GluCUC^ half using the cationic liposome 1,2-dioleoyloxy-3-trimethylammonium-propane (DOTAP) which mimics EVs. As controls, a 20-nt HIV-1-derived ssRNA termed ssRNA40 (**S2 Fig**), known to strongly activate endosomal TLRs [7], and its inactive mutant (ssRNA40-M), in which U is replaced with A, were also transfected. As shown in **Fig 5A**, transfections of the 5′-tRNA^HisGUG^ half and ssRNA40, a positive control, increased the production of TNFα, interleukin (IL)-1β, and IL-12p40 mRNAs, whereas transfections of the 5′-tRNA^GluCUC^ half and ssRNA40-M, a negative control, did not. Induction of the secretion of TNFα and IL-1β into culture medium upon the transfection of the 5′-tRNA^HisGUG^ half, as well as ssRNA40, was further confirmed by ELISA (**Fig 5B**). Transfection of the 5′-tRNA^HisGUG^ half using Lipofectamine reagents did not show such inductions (**S6 Fig**), confirming that the delivery of 5′-tRNA^HisGUG^ half to endosomes, not to the cytoplasm, is necessary for the inductions. The strong activation of endosomal TLR by the DOTAP-fused 5′-tRNA^HisGUG^ half was further observed in PHMDMs. Upon transfection of 5′-tRNA^HisGUG^ half into PHMDMs, increased production of TNFα, IL-1β, and IL-12p40 mRNAs (**Fig 5C**) and enhanced secretion of TNFα and IL-1β (**Fig 5D**) were observed. While the calculation of **Fig 2E** indicated the presence of 25 fmol of EV-5′-tRNA^HisGUG^ half per 1 ml of medium (1 μl of EV solution was obtained from 80 μl of medium), 1.8 fmol of 5′-tRNA^HisGUG^ half per 1 ml of medium was sufficient to observe the activation of endosomal TLRs in PHMDMs (**S7 Fig**), suggesting that physiologically relevant amounts of 5′-tRNA^HisGUG^ half can activate endosomal TLR. Earlier studies have shown that modified nucleotides in tRNAs can affect endosomal TLR activation [57,58]. In the region of the 5′-tRNA^HisGUG^ half, mature tRNA^HisGUG^ contains the following 5 posttranscriptionally modified nucleotides: dihydrouridine (D) at np 16, 19, and 20; peudouridine (Ψ) at np 32, and queuosine (Q) at np 34 [59–61]. Among the 5 modified nucleotides, Q34 has been reported to block ANG-mediated anticodon cleavage [62] and thus would be absent in the 5′-tRNA^HisGUG^ half. The synthetic 5′-tRNA^HisGUG^ half containing the other 4 modified nucleotides (**S2A Fig**) activated endosomal TLRs as strongly as unmodified RNA (**Fig 5E**), suggesting that the endogenous, modified 5′-tRNA^HisGUG^ half would have the activity. Although mature tRNAs have been reported to be incorporated in EVs [19,20], interestingly, the full-length tRNA^HisGUG^ was incapable of stimulating endosomal TLR (**Fig 5F**) possibly due to its rigid secondary and tertiary structures. These results suggest that shortening mature tRNA^HisGUG^ into less-rigid 5′-half molecules by anticodon cleavage is necessary to activate endosomal TLR.

**Fig 6:**
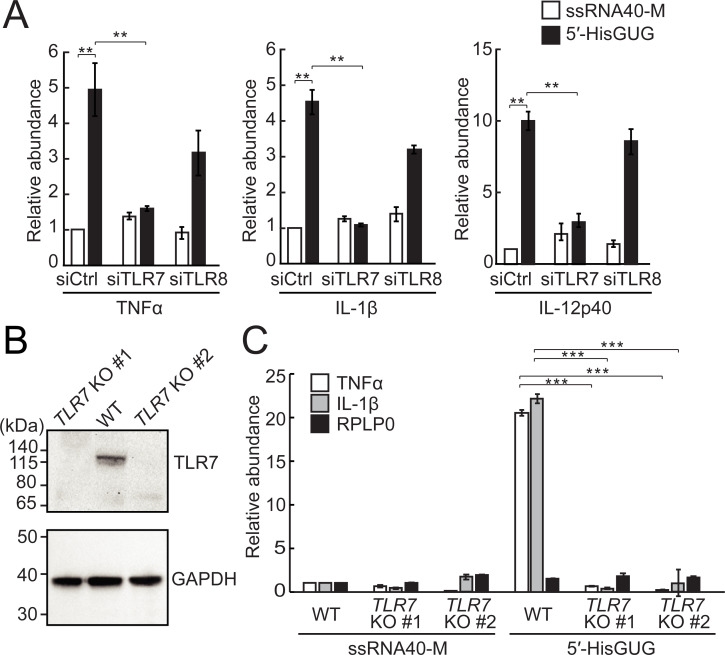
5′-tRNA^HisGUG^ half activates TLR7. **(A)** In HMDMs, the expression of TLR7 or TLR8 was silenced by siRNAs, and then the DOTAP-fused 5′-tRNA^HisGUG^ half or ssRNA40-M was transfected. Total RNAs from the cells were subjected to RT-qPCR for the indicated mRNAs. Averages of 3 experiments with SD values are shown (**P < 0.01; 2-tailed *t* test). **(B)** Lysates from 2 different *TLR7* KO THP-1 cell clones (#1 and #2), as well as from WT cells, were subjected to western blots to confirm the depletion of TLR7 expression. **(C)** The experiments in (A) were performed by using *TLR7* KO cells (***P < 0.001; 2-tailed *t* test). DOTAP, 1,2-dioleoyloxy-3-trimethylammonium-propane; HMDM, human monocyte-derived macrophage; KO, knockout; mRNA, messenger RNA; RT-qPCR, quantitative reverse transcription PCR; SD, standard deviation; TLR, Toll-like receptor; tRNA, transfer tRNA; WT, wild-type.

We next examined whether the 5′-tRNA^HisGUG^ half activates endosomal TLR7 and/or TLR8. siRNA-mediated KD of TLR7 alone or simultaneous KD of TLR7 and TLR8 in HMDMs abolished the up-regulation of TNFα, IL-1β, and IL-12p40 by DOTAP transfection of the 5′-tRNA^HisGUG^ half, whereas TLR8 KD alone did not (**Fig 6A, S8A–S8C Fig**). These results suggest that 5′-tRNA^HisGUG^ half stimulates endosomal TLR7 as strongly as ssRNA40, but not TLR8. To further confirm the involvement of TLR7 in the activity of the 5′-tRNA^HisGUG^ half, by using CRISPR/Cas9 approach, we generated *TLR7* knockout (KO) THP-1 cell lines in which TLR7 expression is completely abolished (**Fig 6B**). The 5′-tRNA^HisGUG^ half did not show the activity to stimulate endosomal TLR in *TLR7* KO cells (**Fig 6C**), confirming that the 5′-tRNA^HisGUG^ half activates endosomal TLR7.

**Fig 7:**
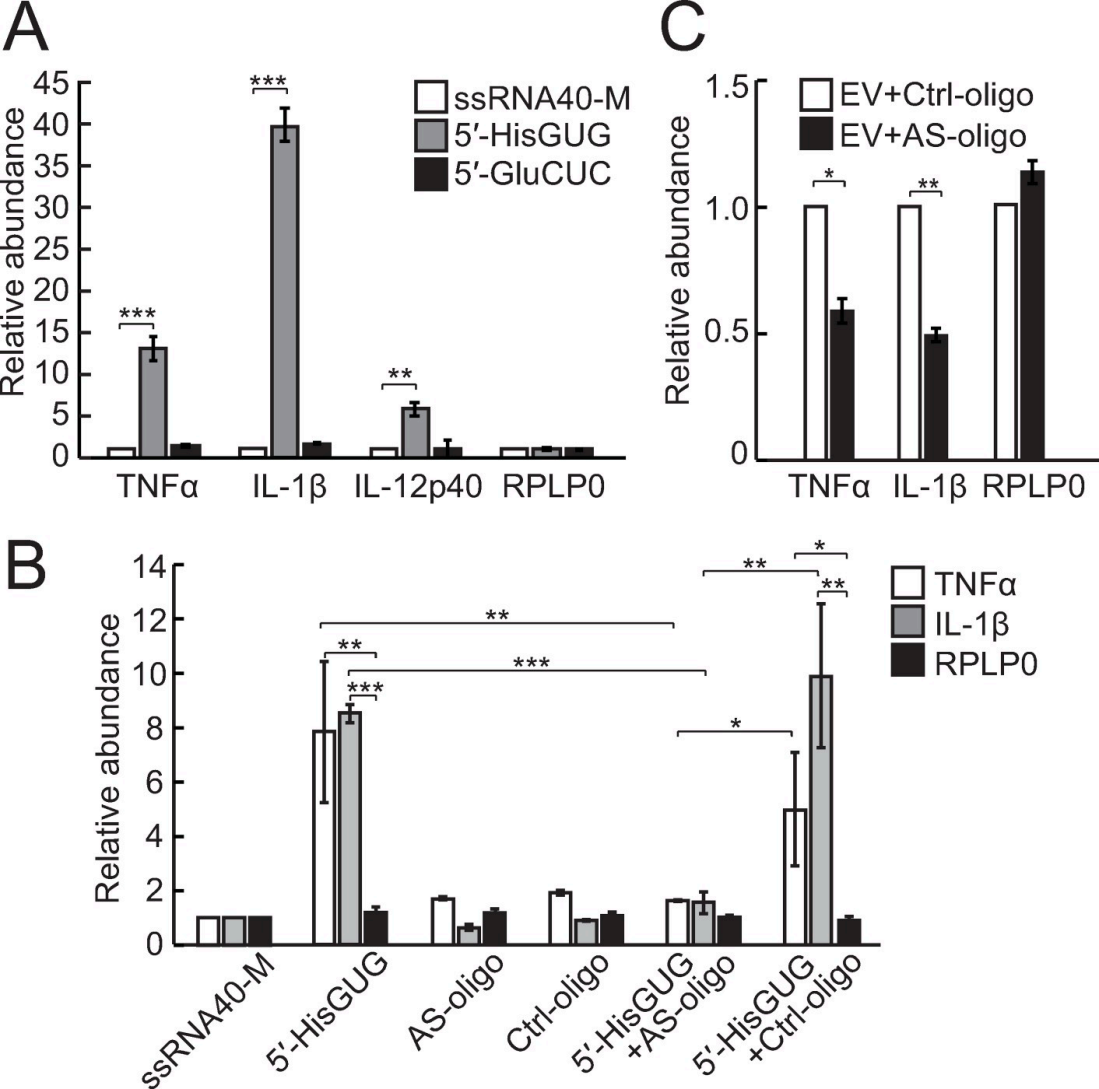
Activation of TLR7 by endogenous EV-5′-tRNA^HisGUG^ half. **(A)** EVs from HMDMs transfected with the indicated 5′-tRNA halves or ssRNA40-M were isolated and applied to recipient HMDMs. Total RNAs from the cells were then subjected to RT-qPCR for the indicated mRNAs. Averages of 3 experiments with SD values are shown (*P < 0.05, **P < 0.01, and ***P < 0.001; 2-tailed *t* test). **(B)** The indicated synthetic RNAs, antisense oligonucleotides of the 5′-tRNA^HisGUG^ half (AS-oligo), the control oligonucleotides with scrambled sequences (Ctrl-oligo), or a mixture (the 5′-tRNA^HisGUG^ half was mixed with an equal amount of the oligonucleotides) were subjected to DOTAP-mediated transfection into HMDMs, and indicated mRNAs were quantified. Averages of 3 experiments with SD values are shown. **(C)** EVs from LPS-treated HMDMs were mixed with DOTAP-fused AS- or Ctrl-oligo and applied to recipient HMDMs. Then, the indicated mRNA expression was quantified. Averages of 3 experiments with SD values are shown. DOTAP, 1,2-dioleoyloxy-3-trimethylammonium-propane; EV, extracellular vehicle; HMDM, human monocyte-derived macrophage; LPS, lipopolysaccharide; mRNA, messenger RNA; RT-qPCR, quantitative reverse transcription PCR; SD, standard deviation; TLR, Toll-like receptor; tRNA, transfer tRNA.

To test whether the EV-5′-tRNA^HisGUG^ half activates TLR7, we transfected the 5′-tRNA^HisGUG^ half, 5′-tRNA^GluCUC^ half, and ssRNA40-M (negative control) into HMDMs, and the EVs isolated from the cells were applied to recipient HMDMs. As shown in **Fig 7A**, EVs isolated from HMDMs that transiently expressed the 5′-tRNA^HisGUG^ half were able to induce immune response. To further confirm the activity of endogenous EV-5′-tRNA^HisGUG^ halves, we utilized antisense oligonucleotides of the 5′-tRNA^HisGUG^ half and control oligonucleotides with scrambled sequences. In a DOTAP transfection experiment, both oligonucleotides did not show activity for endosomal TLR by themselves (**Fig 7B**). When mixed with an equal amount of 5′-tRNA^HisGUG^ half, the antisense oligonucleotides impaired TLR7 activation by the 5′-tRNA^HisGUG^ half, but the control oligonucleotides did not (**Fig 7B**), confirming the antisense oligonucleotides’ activity to block the 5′-tRNA^HisGUG^ half. In the experiment using the EVs isolated from HMDMs, strikingly, the antisense oligonucleotides of the 5′-tRNA^HisGUG^ half reduced the EV-induced up-regulation of TNFα and IL-1β by 40% to 60% (**Fig 7C**). Taken together, these results confirmed that endogenous 5′-tRNA^HisGUG^ halves, which are transferred from EVs to recipient cells, have activity to promote cytokine productions by stimulating endosomal TLR7.

### Levels of circulating 5′-tRNA halves are elevated in the plasma of Mtb-infected patients

**Fig 8:**
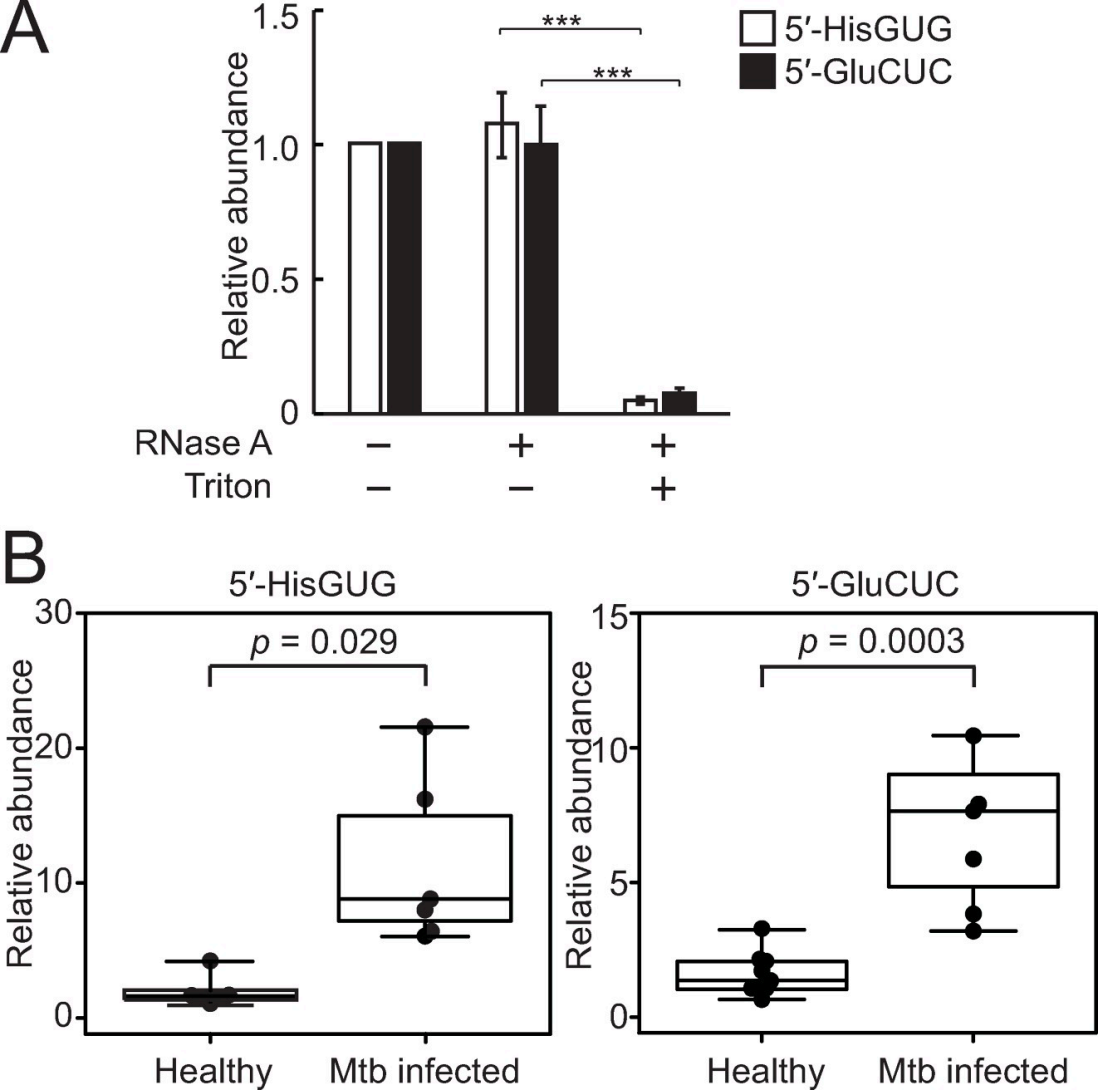
Enhanced accumulation of tRNA halves in Mtb-infected patients. **(A)** Human plasma sample (batch #1) was treated with RNase A and/or Triton X-100 and then subjected to TaqMan RT-qPCR for quantification of 5′-tRNA halves. Averages of 3 experiments with SD values are shown (***P < 0.001; 2-tailed *t* test). **(B)** RNAs isolated from plasma samples of healthy individuals (*n* = 8) or Mtb-infected patients (*n* = 6) were subjected to TaqMan RT-qPCR for the indicated 5′-tRNA halves. The quantified 5′-tRNA half levels were normalized to spike-in synthetic mouse piR-3 levels. Mtb, *Mycobacterium tuberculosis*; RT-qPCR, quantitative reverse transcription PCR; SD, standard deviation; tRNA, transfer tRNA.

We further examined 5′-tRNA half expression in human plasma samples. Plasma EVs were isolated (**S9A Fig**) and subjected to treatments with RNase in the presence or absence of detergent. While the plasma EVs treated with RNase alone yielded similar amplification signals to untreated EVs, the EVs treated with both RNase and detergent yielded drastically reduced amplification signals (**S9B Fig**), confirming the presence of 5′-tRNA halves inside the plasma EVs. Because the quantification of 5′-tRNA halves using plasma RNAs showed similar amplification patterns with no changes in the levels of 5′-tRNA halves upon RNase treatment of plasma (**Fig 8A and S9C Fig**), the detected 5′-tRNA halves in plasma samples were expected to be mostly present inside plasma EVs. We then quantified the 5′-tRNA haves in the plasma samples from healthy individuals or Mtb-infected patients. Because the expression of tRNA halves can be affected by sex hormones [28] and aging [34], we limited the examined individuals to males aged 30 to 35 years. During RNA extraction, a synthetic mouse piRNA was added as a spike-in control, and its abundance was used for normalization. As shown in **Fig 8B**, the expression levels of 2 examined 5′-tRNA halves were markedly enhanced in Mtb-infected patients compared to healthy individuals. The 5′-tRNA^HisGUG^ half in particular was highly elevated at approximately 10-fold higher in Mtb-infected patients than in control individuals. These results suggest that the up-regulation and secretion of 5′-tRNA halves upon infection are not limited to cell culture settings but also occur in actual pathological situations in pathogenic microbe-infected patients.

## Discussion

**Fig 9:**
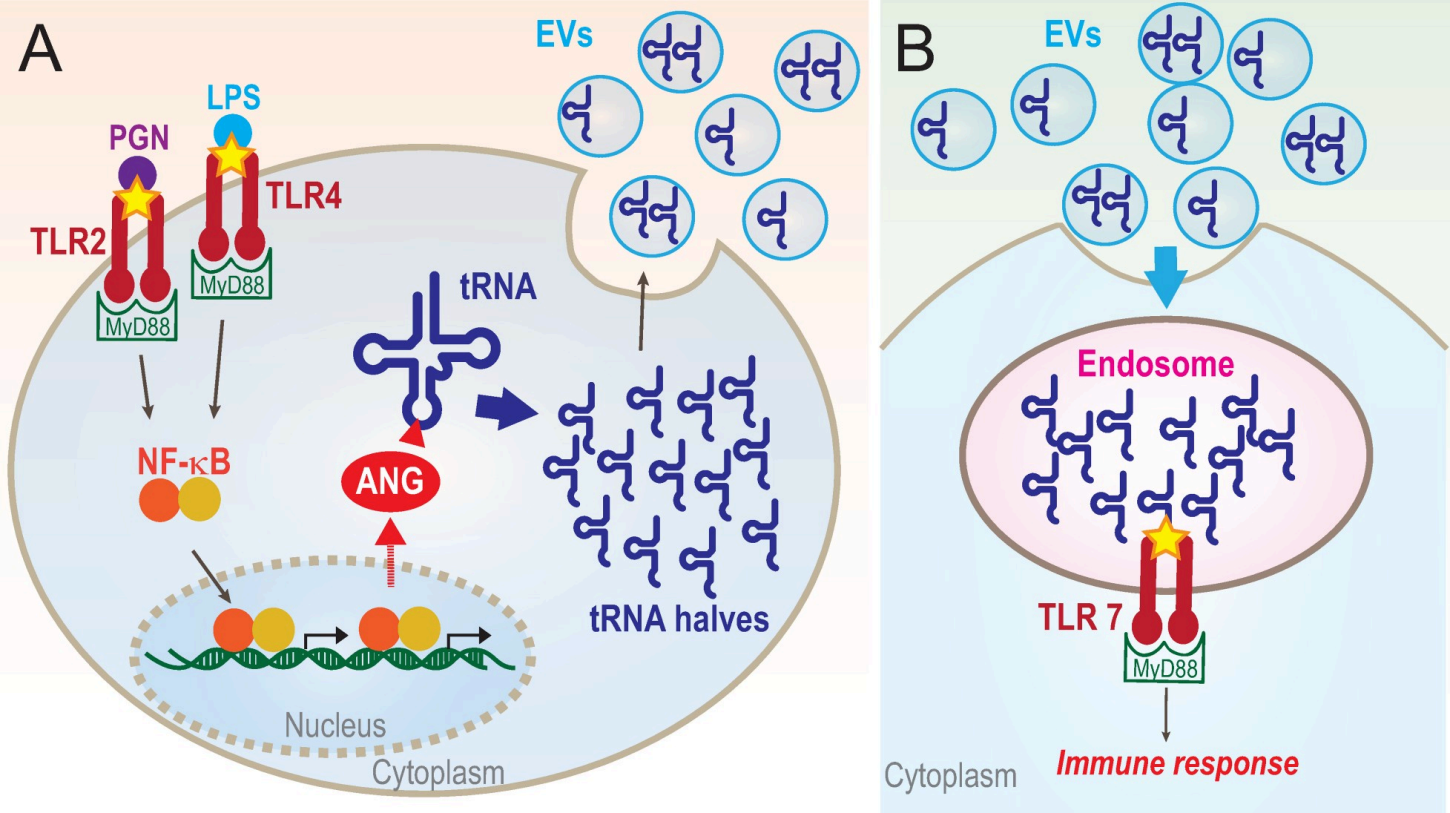
A proposed model for 5′-tRNA half-mediated immune response. **(A)** Surface TLR stimulation culminates in activation of NF-κB, leading to up-regulation of ANG, which cleaves the anticodon loops of tRNAs. The resultant 5′-tRNA halves are secreted by being packaged into EVs and function as signaling molecules. **(B)** EV-5′-tRNA halves are delivered into endosomes in recipient cells and activate TLR7, which promote the immune response. ANG, angiogenin; EV, extracellular vehicle; TLR, Toll-like receptor; tRNA, transfer tRNA.

Here, we identified a novel role of 5′-tRNA halves as activators of TLR7. Both BCG infection and PAMP-mediated surface TLR activation induced the expression of 5′-tRNA halves in HMDMs. Considering the results of earlier studies on the function of 5′-tRNA halves in the stress response, translation, and cell proliferation [28,63–65], infection-induced 5′-tRNA halves could function in various biological processes inside macrophages. In the present study, we focused on the secretion of 5′-tRNA halves into EVs and their role as stimulators of endosomal TLRs in recipient cells. Strikingly, our analyses revealed the abundant accumulation of 5′-tRNA halves in HMDM-secreted EVs and their delivery to endosomes in recipient cells for the activation of TLR7. We propose that infection-induced 5′-tRNA halves function as “immune activators” by being delivered to endosomes in surrounding cells via EV-mediated cell–cell communication and by activating TLR7 (**Fig 9**).

Previous studies have shown that stress stimuli and sex hormone signaling pathways induce ANG-catalyzed cleavage of the anticodon loop of tRNAs, leading to the expression of tRNA halves termed tRNA-derived stress-induced RNAs (tiRNAs) and sex hormone-dependent tRNA-derived RNAs (SHOT-RNAs), respectively [25,26,28]. In tiRNA biogenesis, tRNA cleavage is triggered by decreased levels of RNH1, an ANG inhibitor, which increase ANG availability for tRNA cleavage [66]. Although the mechanism of SHOT-RNA biogenesis is unknown, estrogen or androgen receptors, functioning as transcription factors, might regulate the expression of ANG and/or RNH1. In the case of infection-induced tRNA halves, our analyses revealed that TLR-activated NF-κB up-regulates the expression levels of ANG mRNA, potentially leading to enhanced levels of ANG protein available for tRNA cleavage. If this is the mechanism behind tRNA half production, because not only surface TLR pathways but also the TLR7 pathway culminates in NF-κB activation, there could be a feed-forward loop in which TLR7 activation by 5′-tRNA halves induces the expression of 5′-tRNA halves for further activation of TLR7. In addition, because dysregulation of NF-κB is linked to various diseases, such as cancers and inflammatory and autoimmune diseases [67–69], the potential regulation of tRNA half production by NF-κB suggests the involvement of tRNA halves in such diseases.

By using cP-RNA-seq, we identified the complete expression repertories of 5′-tRNA halves in HMDMs and their secreted EVs, revealing that only specific tRNA species serve as major substrates for infection-induced tRNA half expression. The molecular mechanism underlying the anticodon loop cleavage of specific tRNA species remains unknown. Because major substrate tRNAs such as cyto tRNA^ValCAC^, tRNA^ValAAC^, tRNA^GlyGCC^, tRNA^HisGUG^, and tRNA^GluCUC^ were also identified as major sources of SHOT-RNAs in human breast cancer cells [28], those tRNAs may be universally susceptible to ANG cleavage, or the molecular factors determining the susceptibility of tRNAs to anticodon cleavage, such as tRNA modifications, may be regulated similarly between the biogenesis of sex hormone–and infection-induced tRNA halves. The difference in the expression profiles of 5′-tRNA halves between HMDMs and their secreted EVs suggests selective packaging of 5′-tRNA halves into EVs. Selective packaging of the 5′-tRNA^HisGUG^ half into EVs is intriguing as this half is highly active in TLR7 stimulation. Although the mechanism of EV RNA content selection is unknown, biased EV incorporation has been also shown for miRNAs [70–72] and tRFs [73,74]. Because Y-box protein 1 (YBX1) has been reported to interact with 5′-tRNA halves [64] and has been implicated in the sorting of miRNAs for packaging into EVs [72], such RNA-binding proteins could be involved in the selective packaging of 5′-tRNA halves. Among the cellular 5′-tRNA^HisGUG^ half species, only a specific 5′-tRNA^HisGUG^ half, from G_1_ to G_34_, is preferentially packaged into EVs. Specific sequences and/or secondary/tertiary structures may contribute to preferential binding to RNA-binding proteins responsible for EV packaging. Indeed, in the case of miRNAs, specific 3′-terminal sequences are required to interact with heterogeneous nuclear ribonucleoprotein A2/B1 for preferential incorporation into EVs [70].

One of the most remarkable characteristics of 5′-tRNA halves is their abundance. Although miR-150 was identified as one of the most abundant miRNAs in HMDMs and their EVs [44], the present quantification revealed the abundance of the 5′-tRNA^HisGUG^ half in HMDMs and EVs to be over 130-fold and 210-fold higher, respectively. Although miRNAs have been shown to function as ligands for TLR7, considering ligand–receptor interactions, 5′-tRNA halves with much more abundance could be more efficient, superior TLR ligands than miRNAs. Given that T4 PNK treatment greatly enhanced amounts of EV-cDNAs during our sequencing procedure, it is predicted that EV-short ncRNA species are mostly 3′-P- or cP-containing RNAs, such as 5′-tRNA halves, and that 3′-OH-containing RNAs, such as miRNAs, are minor species. While studies on EVs have established the role of EV-RNAs as cell–cell communication agents [75], most current studies rely on standard RNA-seq, which cannot capture the 3′-P or cP-containing RNAs that account for the majority of short RNA species in EVs. Our results suggest the necessity of shedding light on these previously unrecognized RNAs by pretreating EV-RNA fractions with T4 PNK in sequencing studies. Giraldez and colleagues revealed previously unexplored mRNA and lncRNA fragments by phosphor-RNA-seq whose procedure includes T4 PNK treatment [76].

Another striking feature of the 5′-tRNA^HisGUG^ half is its ability to strongly activate TLR7, but not TLR8. This selective activity for TLR7 might result from the high sensitivity of TLR7 to GU-rich ssRNAs, such as the 5′-tRNA^HisGUG^ half, while TLR8 senses AU-rich ssRNAs [77]. The activation of TLR7 by the 5′-tRNA^HisGUG^ half is as high as that by SSRNA40, suggesting the role of the 5′-tRNA^HisGUG^ half as an endogenous ligand for TLR7 with the full capacity to produce an immune response. On the other hand, the 5′-tRNA^GluCUC^ half did not activate TLR7. Because the 5′-tRNA^GluCUC^ half and the 5′-tRNA^HisGUG^ half were similarly delivered to recipient endosomes in our delivery experiments, the inactivity of the 5′-tRNA^GluCUC^ half is probably due to its inefficient binding to TLR7. The lack of 3′-terminal GU-rich sequences may be one of the reasons for the inefficient activity of 5′-tRNA^GluCUC^ toward TLR7 as previous study showed significance of 3′-terminal GU sequences in let-7 miRNA for TLR7 activation [10]. Intriguingly, unlike the 5′-tRNA^HisGUG^ half, the full-length tRNA^HisGUG^ is incapable of activating TLR7, suggesting the cruciality of tRNA cleavage and production of tRNA half molecules to yield active ligands for TLR7.

Finally, we showed the elevation of 5′-tRNA half levels in the plasma of Mtb-infected patients, demonstrating the expressional induction and secretion of 5′-tRNA halves in actual pathological situations. Because up-regulation of 5′-tRNA half expression has been reported upon infection with respiratory syncytial virus [78,79], *Rickettsia* [80], and hepatitis B and C viruses [81], induction of 5′-tRNA halves could be a universal phenomenon among infectious diseases. Considering the expressional differences and the demonstrated roles of 5′-tRNA halves in the innate immune response, further characterization of 5′-tRNA halves may lead to the use of 5′-tRNA halves as potential target candidates for future therapeutic applications and/or circulating biomarkers for noninvasive testing to estimate the severity of infectious diseases and the status of the immune response.

## Materials and methods

### Ethical approval

The Office of Human Research (OHR) of Thomas Jefferson University (TJU) approved our use of patient samples without private information in accordance with all federal, institutional, and ethical guidelines (#OHR-19: Expressions of noncoding RNAs in human plasma and serum samples). We obtained the plasma samples from a company BioIVT (Westbury, New York, United States of America) without receiving patients’ information.

### Cell culture, BCG infection, PAMP treatment, and NF-κB inhibition

THP-1 human acute monocytic leukemia cells (American Type Culture Collection, Manassas, Virginia, USA) were cultured in RPMI 1640 medium (Corning, Corning, New York, USA) and differentiated into HMDMs using phorbol 12-myristate 13-acetate (PMA; Sigma-Aldrich, St. Louis, Missouri, USA) as described previously [82,83]. Human CD14+ monocytes (Precision for Medicine, New Jersey, USA) were cultured in Gibco SFM medium (Thermo Fisher Scientific, Waltham, Massachusetts, USA) and differentiated into PHMDMs using macrophage colony-stimulating factor (M-CSF; Tonbo Biosciences, San Diego, California, USA) as described previously [84]. HMDMs were infected with viable or HK *M*. *bovis* BCG (DSMZ, Braunschweig, Germany) as described previously [82,83]. Zero viability of HK-BCG was confirmed by spading its suspension on Middlebrook 7H11 agar plates supplemented with OADC and confirming the absence of colonies in at least 3 weeks. For activation of surface TLRs, HMDMs and PHMDMs were cultured with medium containing 100 ng/ml of LPS from *Escherichia coli* O111:B4 (Sigma-Aldrich) or PGN from *Bacillus subtilis* (Sigma-Aldrich) for 12 h. For inhibition of NF-κB, HMDMs were treated with 40 μM of JSH-23 (Sigma-Aldrich) for 24 h.

### EV isolation

EVs were isolated from the culture medium of LPS-treated HMDMs according to an ultracentrifugation-based method described previously [44]. In brief, dead cells and cell debris in the culture medium were removed by successive centrifugation at 300 g for 10 min, 2,000 g for 10 min, and 10,000 g for 30 min. The supernatant was then ultracentrifuged using Sorvall WX+ Ultracentrifuge Series (Thermo Fisher Scientific) at 110,000 g for 2 h. The pellet was washed with PBS and ultracentrifuged again at 110,000 g for 2 h to eliminate contaminant proteins. The final pellet was collected as the EV fraction. The data regarding EV isolation and characterization are available in EV-TRACK database (EV-TRACK ID: EV190062) [85]. To confirm the presence of EV-RNAs, the isolated EVs were incubated with PureLink RNase A (200 ng/μl, Thermo Fisher Scientific) with or without 0.1% Triton X-100 at 37°C for 30 min.

### NTA and transmission electron microscopy

Size distributions of the isolated EVs were analyzed by NTA using NanoSight NS300 (Malvern Analytical, Malvern, United Kingdom), as described previously [86], at the Flow Cytometry Facility of the Sidney Kimmel Cancer Center at TJU. The isolated EVs were further visualized by transmission electron microscopy (JEOL, Akishima, Tokyo, Japan) at the Centralized Research Facilities at Drexel University.

### Quantification of RNAs by TaqMan RT-qPCR, stem-loop RT-qPCR, and standard RT-qPCR

Total RNA from the cells and EVs was isolated using TRIsure (Bioline, Swedesboro, New Jersey, USA). TaqMan RT-qPCR for specific quantification of 5′-tRNA halves was performed according to our previously described tRNA half quantification method [28]. Briefly, to remove cP from 5′-tRNA halves, total RNA was treated with T4 PNK, followed by ligation to a 3′-RNA adapter by T4 RNA ligase. Ligated RNA was then subjected to TaqMan RT-qPCR using the One Step PrimeScript RT-PCR Kit (Takara Bio, Kusatsu, Shiga, Japan), 200 nM of a TaqMan probe targeting the boundary of the target RNA and the 3′-adapter, and forward and reverse primers. The TaqMan probe and primer sequences are shown in **S2 Table**. Stem-loop RT-qPCR for quantification of miRNAs and piRNAs was performed as previously described [87,88]. In brief, total RNA was treated with DNase I (Promega, Madison, Wisconsin, USA) and subjected to reverse transcription using SuperScript III reverse transcriptase (Thermo Fisher Scientific) and a stem-loop reverse primer. The synthesized cDNAs were then subjected to PCR using SsoFast Evagreen Supermix (Bio-Rad, Hercules, California, USA) and forward and reverse primers. Sequences of the primers used are shown in **S3 Table**. Standard RT-qPCR was used for quantification of mRNAs. Briefly, DNase I-treated total RNA was subjected to reverse transcription using RevertAid Reverse Transcriptase (Thermo Fisher Scientific) and a reverse primer. The synthesized cDNAs were then subjected to PCR using 2×qPCR Master Mix (Bioland Scientific, Paramount, California, USA) and forward and reverse primers. Sequences of the primers used are shown in **S4 Table**.

### Northern blot

Northern blot was performed as previously described [28] with the following antisense probes: 5′-tRNA^HisGUG^ half, 5′-CAGAGTACTAACCACTATACGATCACGGC-3′; 5′-tRNA^GluCUC^ half, 5′-GCGCCGAATCCTAACCACT-3′; and miR-16, 5′-GCCAATATTTACGTGCTGCTA-3′.

### Western blot

Western blot was performed as described previously [87]. Lysates of HMDMs or their EVs were prepared in RIPA buffer supplemented with cOmplete Protease Inhibitor Cocktail (Roche, Basel, Switzerland). Anti-Alix (1A12, Santa Cruz Biotechnology, Dallas, Texas, USA), anti-CD63 (Santa Cruz Biotechnology), anti-Calnexin (AF18, Santa Cruz Biotechnology), anti-cytochrome c (A-8, Santa Cruz Biotechnology), and anti-TLR7 (4F4, sc-57463, Santa Cruz Biotechnology) were used as primary antibodies.

### cP-RNA-seq and bioinformatics

For cP-RNA-seq, 25–50-nt RNAs were gel-purified from the total RNA of LPS-treated HMDMs and subjected to the cP-RNA-seq procedure as previously described [28,30,32–34]. For EV-5′-tRNA half sequencing, EV-RNA was first treated with T4 PNK to remove cP from the 5′-tRNA halves, followed by adapter ligation and cDNA amplification using the TruSeq Small RNA Sample Prep Kit (Illumina, San Diego, California, USA). The amplified cDNAs were gel-purified and sequenced using the Illumina NextSeq 500 system at the MetaOmics Core Facility of the Sidney Kimmel Cancer Center at TJU. The sequence libraries contain approximately 35 to 44 million raw reads (**S1 Table**) and are publicly available from the NCBI Sequence Read Archive (accession no. SRR8430192, SRR8430191, and SRR8430193). Bioinformatic analyses were performed as described previously [33,34]. Reads were mapped to 471 mature cyto tRNAs obtained from GtRNAdb [51], and then to mature rRNAs, to mRNAs of RefSeq with NM-staring accession numbers (NM is an accession prefix of known RefSeq), to the mitochondrial genome (GenBank: CM001971.1 sequence plus 22 mitochondrial tRNA sequences), and to the whole genome (GRCh37/hg19).

### In vitro RNA synthesis

The synthetic RNAs used in this study are shown in **S5 Table**. While antisense oligonucleotides, miRNAs, and a piRNA (spike-in) were synthesized by Integrated DNA Technologies, 5′-tRNA halves, FL-tRNA^HisGUG^, and ssRNA40 were synthesized by an in vitro reaction as described previously [53]. dsDNA templates were synthesized using PrimeSTAR GXL DNA Polymerase (Takara Bio) and the primers shown in **S6 Table**. The templates were then subjected to an in vitro transcription reaction with T7 RNA polymerase (New England Biolabs, Ipswich, Massachusetts, USA) at 37°C for 4 h. For 5′-tRNA^GluCUC^ half production, the in vitro synthesized RNA contained the ribozyme sequence to generate a mature 5′-end as described previously [89], so the reaction mixture was further incubated for 3 cycles at 90°C for 2.5 min and 37°C for 15 min, allowing the ribozyme reaction. The synthesized RNAs were then gel-purified using denaturing PAGE with single-nucleotide resolution, and the quality of the gel-purified RNAs was confirmed by denaturing PAGE as shown in **S2B Fig**. For FL-tRNA^HisGUG^, we performed annealing by incubating it in the annealing buffer consisting of 50 mM Tris-HCl (pH 8) and 100 mM MgCl_2_ at 70°C for 3 min, followed by incubation at 37°C for 20 min. Low Molecular Weight Marker 10 to 100 nt (Affymetrix, Santa Clara, California, USA) was used as a marker in the denaturing PAGE.

### Fluorescent labeling of 5′-tRNA halves and their EV-mediated delivery to cells

The synthetic 5′-tRNA^HisGUG^ half and 5′-tRNA^GluCUC^ half were fluorescent-labeled at their 3′-end based on a previously described method [54]. In brief, synthetic RNAs were incubated in 100 mM NaOAc (pH 5.2) and 100 μM NaIO_4_ at room temperature for 90 min, followed by ethanol precipitation. Then the pellet was dissolved in a solution containing 1.5 mM FTSC (Cayman Chemical, Ann Arbor, Michigan, USA) and 100 mM NaOAc (pH 5.2), followed by overnight incubation at 4°C. After ethanol precipitation, the labeled RNAs were subjected to Centri-Spin 10 (Princeton Separations, Adelphia, New Jersey, USA) purification to remove unreacted FTSC. Then, 80 pmol of the labeled RNA was transfected into HMDMs using RNAiMAX (Thermo Fisher Scientific). After 24 h, the cells were washed with PBS and further incubated for 12 h with LPS, and the cell culture medium was subjected to EV isolation as described above. The isolated EV fraction was then added to HMDMs, followed by incubation for 6 h, and visualization of the labeled 5′-tRNA halves with Rab7 and TLR7 by confocal microscopy as described below.

### Immunofluorescence staining and confocal microscopy

Immunofluorescence staining was performed as described previously [87] using anti-Rab7 (diluted 1:100, Cell Signaling Technology, Danvers, Massachusetts, USA), anti-TLR7 (diluted 1:500, Novus Biologicals, Littleton, Colorado, USA), and Alexa Fluor 488 goat anti-rabbit IgG (diluted 1:2000, Thermo Fisher Scientific) as primary and secondary antibodies, respectively. After DNA counterstaining with ProLong Gold Antifade Reagent with DAPI (Thermo Fisher Scientific), images were acquired using a Nikon Eclipse Ti-U confocal microscope (Melville, New York, USA) at the Bioimaging Facility of the Sidney Kimmel Cancer Center at TJU.

### DOTAP-mediated RNA delivery to endosomes

To deliver RNAs to endosomes, we used DOTAP liposomal transfection reagent (Sigma-Aldrich) as previously described [11,56]. In brief, 230 pmol or other various amounts of synthetic RNAs were mixed with 60 μl of HBS buffer and 15 μl of DOTAP reagent and incubated for 15 min. The RNA-DOTAP solution was then added to 1 ml HMDM or PHMDM medium, followed by incubation of the cells for 16 h.

### EV-mediated RNA delivery to endosomes

Synthetic 5′-tRNA^HisGUG^ half, 5′-tRNA^GluCUC^ half, and ssRNA40-M (80 pmol) were transfected to HMDMs (9 × 10^6^ cells) using RNAiMAX (Thermo Fisher Scientific). After 24 h, the cells were washed with PBS and further incubated for 12 h, and the cell culture medium was subjected to EV isolation as described above. The isolated EVs were then added to HMDMs (1 × 10^6^ cells), followed by incubation for 12 h, RNA extraction, and RT-qPCR quantification of TNFα, IL-1β, and IL-12p40 mRNAs.

Regarding experiments using antisense oligonucleotides, control oligonucleotides with scrambled sequences or antisense oligonucleotides for the 5′-tRNA^HisGUG^ half (**S5 Table**) were first infused with DOTAP as described above. EVs isolated from LPS-treated HMDMs were mixed with the DOTAP–oligonucleotides solution and then were applied to recipient HMDMs, followed by incubation for 16 h. To eliminate possible effects of potential endotoxin (LPS) contamination, EVs isolated from LPS-treated HMDMs were incubated with 10 mg/ml polymyxin B (PMB) (Sigma-Aldrich) at 4°C for 1 h prior to mixing with the DOTAP–oligonucleotides solution.

### ELISA

For ELISA experiment, RNA transfection using DOTAP was performed in Opti-MEM (Thermo Fisher Scientific), and the culture medium from 1 × 10^6^ HMDMs or 1 × 10^5^ PHMDMs was subjected to ELISA (R&D Systems, Minneapolis, Minnesota, USA) for quantification of TNFα and IL-1β. Their absolute amounts were calculated based on standard curves.

### RNAi KD of ANG, TLR7, and TLR8

To silence the expression of ANG, TLR7, and TLR8, siRNAs designed in previous reports [28,56,90] were synthesized by Bioland Scientific. Their sense strand sequences are 5′-AAACCUAAGAAUAAGCAAGUCAU-3′, 5′-GCCUUGAGGCCAACAACAUUU-3′, and 5′-GGUGGUGCUUCAAUUAAUAUU-3′ for ANG, TLR7, and TLR8, respectively. ON-TARGETplus Nontargeting siRNA #2 (D-001810-02, Dharmacon, Lafayette, Colorado, USA) was used as a negative control as previously described [28]. HMDMs were transfected with 50 nM of each siRNA using RNAiMAX (Thermo Fisher Scientific). In simultaneous KD of TL7 and TLR8, 100 nM of the siRNA mixture for TLR7/8 (50 nM each for TLR7 and TLR8) and 100 nM of control siRNA were used. In 60 h after transfection, LPS were added and HMDMs were further incubated for 12 h.

### *TLR7* KO THP-1 cell lines

*TLR7* KO THP-1 cells were generated using the CRISPR/Cas9 system at Genome Editing Institute in ChristianaCare. Two different clones, KO #1 and KO #2, were generated using gRNA1 (5′-ACUUUCAGGUGUUUCCAAUG-3′) and gRNA2 (5′-UAGGAAACCAUCUAGCCCCA-3′), respectively. The KO cells were differentiated into HMDMs and used for transfection of DOTAP-fused RNAs as described above. Confirmation of TLR7 depletion in the KO cells was done using western blot analysis as described above.

### Human plasma samples and RNA isolation

Human plasma samples were derived from healthy or Mtb-infected males aged 30 to 35 years and obtained from BioIVT. For RNA isolation, 500 μl of plasma was first centrifuged at 16,060 g for 5 min, then 400 μl of supernatant was mixed with synthetic mouse piR-3 spike-in control (20 fmol) and subjected to RNA extraction using TRIzol LS (Thermo Fisher Scientific). The extracted RNAs were further subjected to purification using the miRNeasy Mini Kit (Qiagen, Hilden, Germany). Based on the quantification of miR-451 and miR-23a-3p and calculation of “miR ratio” as described earlier [91], no hemolysis was observed in any of the plasma samples. The extracted RNA samples were subjected to quantification of 5′-tRNA halves, and quantification of piR-3 (spike-in) was used for normalization.

## Supporting information

S1 Fig NF-κB-mediated up-regulation of the expression of ANG mRNA upon surface TLR activation. **(A)** HMDMs were transfected with control siRNA (siControl) or siRNA targeting ANG (siANG) and incubated for 60 h. LPS was then added, and the cells were further cultured for 12 h. RT-qPCR confirmed the reduction of ANG mRNA upon siANG transfection (RPLP0: control). Averages of 3 experiments with SD values are shown (*P < 0.05, **P < 0.01, and ***P < 0.001; 2-tailed *t* test). **(B)** After siRNA transfection and LPS treatment of HMDMs, RNAs isolated from the HMDMs were subjected to quantification of 5′-tRNA halves. Averages of 3 experiments with SD values are shown. **(C, D)** Total RNAs from HMDMs (A) or PHMDMs (B), treated with LPS or PGN, were subjected to RT-qPCR for ANG and RPLP0 (control) mRNAs. HMDMs/PHMDMs without treatment served as a control. Averages of 3 experiments with SD values are shown. **(E)** Alignment patterns of ChIP-seq reads [41] around the *ANG* gene region (14q11.2: 21,152–21,162 kb) for the indicated NF-κB family proteins. The Integrative Genomics Viewer was used for visualization. **(F, G)** Total RNAs from HMDMs treated with LPS alone or LPS and JSH-23 (a NF-κB inhibitor) were subjected to RT-qPCR for the indicated mRNAs. ANG, angiogenin; ChIP-seq, chromatin immunoprecipitation and sequencing; HMDM, human monocyte-derived macrophage; LPS, lipopolysaccharide; mRNA, messenger RNA; PGN, peptidoglycan; PHMDM, primary human monocyte-derived macrophage; RT-qPCR, quantitative reverse transcription PCR; SD, standard deviation; TLR, Toll-like receptor; tRNA, transfer tRNA.
(EPS)

S2 Fig Synthetic RNAs used in the present study. **(A)** Synthetic RNA sequences. Guanosine and uridine are shown in red circles. Modified nucleotides [dihydrouridine (D) and peudouridine (Ψ)] are shown in green and blue circles, respectively. **(B)** Indicated synthetic RNAs were synthesized by in vitro transcription, gel-purified, and analyzed with denaturing PAGE.
(EPS)

S3 Fig Standard curves for the quantification of miR-150 and 5′-tRNA^HisGUG^ half. Indicated amounts of synthetic RNAs were subjected to stem-loop/TaqMan RT-qPCRs. Proportional correlations of synthetic RNA input to the Ct were observed and used as standard curves for estimation of the expression levels of respective RNAs. Ct, cycle threshold; RT-qPCR, quantitative reverse transcription PCR; tRNA, transfer tRNA.
(EPS)

S4 Fig tRNA anticodon cleavage sites for generation of 5′-tRNA halves. Cleavage sites in the tRNA anticodon loops were predicted based on the 3′-terminal positions of the 5′-tRNA halves. Anticodons are shown in green. tRNA, transfer tRNA.
(EPS)

S5 Fig Delivery of EV-5′-tRNA halves into endosomal TLR7. **(A, B)** Florescent end-labeled, synthetic 5′-tRNA^HisGUG^ half (A) or 5′-tRNA^GluCUC^ half (B) was transfected into HMDMs and observed in green. Scale bar, 20 μm. **(C, D)** EVs produced from host HMDMs containing the labeled 5′-tRNA^HisGUG^ half or 5′-tRNA^GluCUC^ half were isolated and applied to recipient HMDMs. Delivery of the labeled, EV-5′-tRNA^HisGUG^ half (C) or EV-5′-tRNA^GluCUC^ half (D) into endosomes was observed in green. Immunofluorescence staining of TLR7 is shown in red, and DNA was counterstained with DAPI in blue. Scale bar, 100 μm. Clear co-localization of the labeled 5′-tRNA halves and TLR7 was observed. EV, extracellular vehicle; HMDM, human monocyte-derived macrophage; TLR, Toll-like receptor; tRNA, transfer tRNA.
(TIF)

S6 Fig Lipofectamine-mediated transfection of 5′-tRNA^HisGUG^ half has no effect on immune response. Using RNAiMAX or Lipofectamine LTX (Thermo Fisher Scientific), the synthetic 5′-tRNA^HisGUG^ half and ssRNA40 were transfected into HMDMs. Total RNAs from the cells were subjected to RT-qPCR for the indicated mRNAs. Averages of 3 experiments with SD values are shown. HMDM, human monocyte-derived macrophage; mRNA, messenger RNA; RT-qPCR, quantitative reverse transcription PCR; SD, standard deviation; tRNA, transfer tRNA.
(EPS)

S7 Fig Activation of endosomal TLRs by various amounts of 5′-tRNA^HisGUG^ half. **(A)** The indicated amounts of the synthetic 5′-tRNA^HisGUG^ half were transfected into HMDMs using DOTAP. Total RNAs from the cells were subjected to RT-qPCR for the indicated mRNAs. Averages of 3 experiments with SD values are shown. **(B)** After the RNA transfection, culture medium was subjected to ELISA for quantification of TNFα and IL-1β. DOTAP, 1,2-dioleoyloxy-3-trimethylammonium-propane; HMDM, human monocyte-derived macrophage; IL, interleukin; mRNA, messenger RNA; RT-qPCR, quantitative reverse transcription PCR; SD, standard deviation; TLR, Toll-like receptor; TNFα, tumor necrosis factor α; tRNA, transfer tRNA.
(EPS)

S8 Fig siRNA-mediated KD of TLR7 and TLR8 in HMDMs. **(A)** HMDMs were transfected with control siRNA (siControl) or siRNA targeting TLR7 (siTLR7) or TLR8 (siTLR8). To confirm the reduction of the targeted mRNA, total RNAs from the cells were subjected to RT-qPCR for TLR7 and TLR8 mRNAs (RPLP0: control). **(B)** Double KDs of TLR7 and TLR8 were performed by simultaneously transfecting both siTLR7 and siTLR8, and reduction of the both mRNAs was confirmed by RT-qPCR. **(C)** In HMDMs, the expression of both TLR7 and TLR8 was silenced by siRNAs and then DOTAP-fused 5′-tRNA^HisGUG^ half or ssRNA40-M was transfected. Total RNAs from the cells were subjected to RT-qPCR for the indicated mRNAs. DOTAP, 1,2-dioleoyloxy-3-trimethylammonium-propane; HMDM, human monocyte-derived macrophage; KD, knockdown; mRNA, messenger RNA; RT-qPCR, quantitative reverse transcription PCR; TLR, Toll-like receptor.
(EPS)

S9 Fig Detection of tRNA halves in EVs isolated from human plasma. **(A)** EVs were isolated from human plasma and were analyzed by NTA. Representative size distribution profile is shown. **(B)** Isolated EVs were treated with RNase A and/or Triton X-100 and then subjected to TaqMan RT-qPCR for quantification of 5′-tRNA halves. Averages of 3 experiments with SD values are shown (*P < 0.05, **P < 0.01, and ***P < 0.001; 2-tailed *t* test). **(C)** Human plasma samples (batches #2–4) were treated with RNase A and/or Triton X-100 and then subjected to TaqMan RT-qPCR for quantification of 5′-tRNA halves. Averages of 3 experiments with SD values are shown. EV, extracellular vehicle; NTA, nanoparticle tracking analysis; RT-qPCR, quantitative reverse transcription PCR; SD, standard deviation; tRNA, transfer tRNA.
(EPS)

S1 Movie NTA analysis from control (PBS) sample. NTA, nanoparticle tracking analysis.
(MP4)

S2 Movie NTA analysis from EV sample. EV, extracellular vehicle; NTA, nanoparticle tracking analysis.
(MP4)

S1 Table Read numbers of sequence libraries. (PDF)

S2 Table Sequences of TaqMan probes and primers for TaqMan RT-qPCR. RT-qPCR, quantitative reverse transcription PCR.
(PDF)

S3 Table Sequences of primers for stem-loop RT-qPCR. RT-qPCR, quantitative reverse transcription PCR.
(PDF)

S4 Table Sequences of primers for standard RT-qPCR. RT-qPCR, quantitative reverse transcription PCR.
(PDF)

S5 Table Sequences of synthetic RNAs/DNAs. (PDF)

S6 Table Sequences of primers for the synthesis of dsDNA templates. (PDF)

S1 Raw Image Original gel images contained in this manuscript, related to Figs 1D, 2A, 3A and 3B. (TIF)

S2 Raw Image Original gel images contained in this manuscript, related to Fig 6B and S2B Fig. (TIF)

S1 Data Numerical data underlying Fig 1A–1C, 1E and 1F; Fig 2B and 2D; Fig 3C–3G; Fig 5A–5F; Fig 6A and 6C; Fig 7A–7C; Fig 8A and 8B; and S1A–S1D and S1F and S1G Fig; S3 Fig; S6 Fig; S7 Fig; S8A–S8C Fig and S9A–S9C Fig. (XLSX)

## Abbreviations

ANG: angiogenin
BCG: *Mycobacterium bovis* BCG
ChIP-seq: chromatin immunoprecipitation and sequencing
cP: 2′,3′-cyclic phosphate
cP-RNA: cP-containing RNA
cyto: cytoplasmi
DOTAP: 1,2-dioleoyloxy-3-trimethylammonium-propane
EV: extracellular vehicle
FTSC: fluorescein-5-thiosemicarbazide
HMDM: human monocyte-derived macrophage
IL: interleukin
KD: knockdown
KO: knockout
lncRNA: long noncoding RNA
LPS: lipopolysaccharide
M-CSF: macrophage colony-stimulating factor
miRNA: microRNA
mRNA: messenger RNA
Mtb: *Mycobacterium tuberculosis*
ncRNA: noncoding RNA
np: nucleotide position
nt: nucleotides
NTA: nanoparticle tracking analysis
OHR: Office of Human Research
PAMP: pathogen-associated molecular pattern
PGN: peptidoglycan
PHMDM: primary human monocyte-derived macrophage
piRNA: Piwi-interacting RNA
PMA: phorbol 12-myristate 13-acetate
RNA-seq: RNA sequencing
RT-qPCR: quantitative reverse transcription PCR
SHOT-RNA: sex hormone-dependent tRNA-derived RNA
snoRNA: small nucleolar RNA
ssRNA: single-stranded RNA
T4 PNK: T4 polynucleotide kinase
tiRNA: tRNA-derived stress-induced RNA
TJU: Thomas Jefferson University
TLR: Toll-like receptor
TNFα: tumor necrosis factor α
tRF: tRNA-derived fragment
tRNA: transfer tRNA
YBX1: Y-box protein 1
