# Supplementary figures and images for: Infection-induced 5′-half molecules of tRNAHisGUG activate Toll-like receptor 7
Source: PLoS Biol. 2020 Dec 17;18(12):e3000982. doi: 10.1371/journal.pbio.3000982 (PMC7745994; doi:10.1371/journal.pbio.3000982)

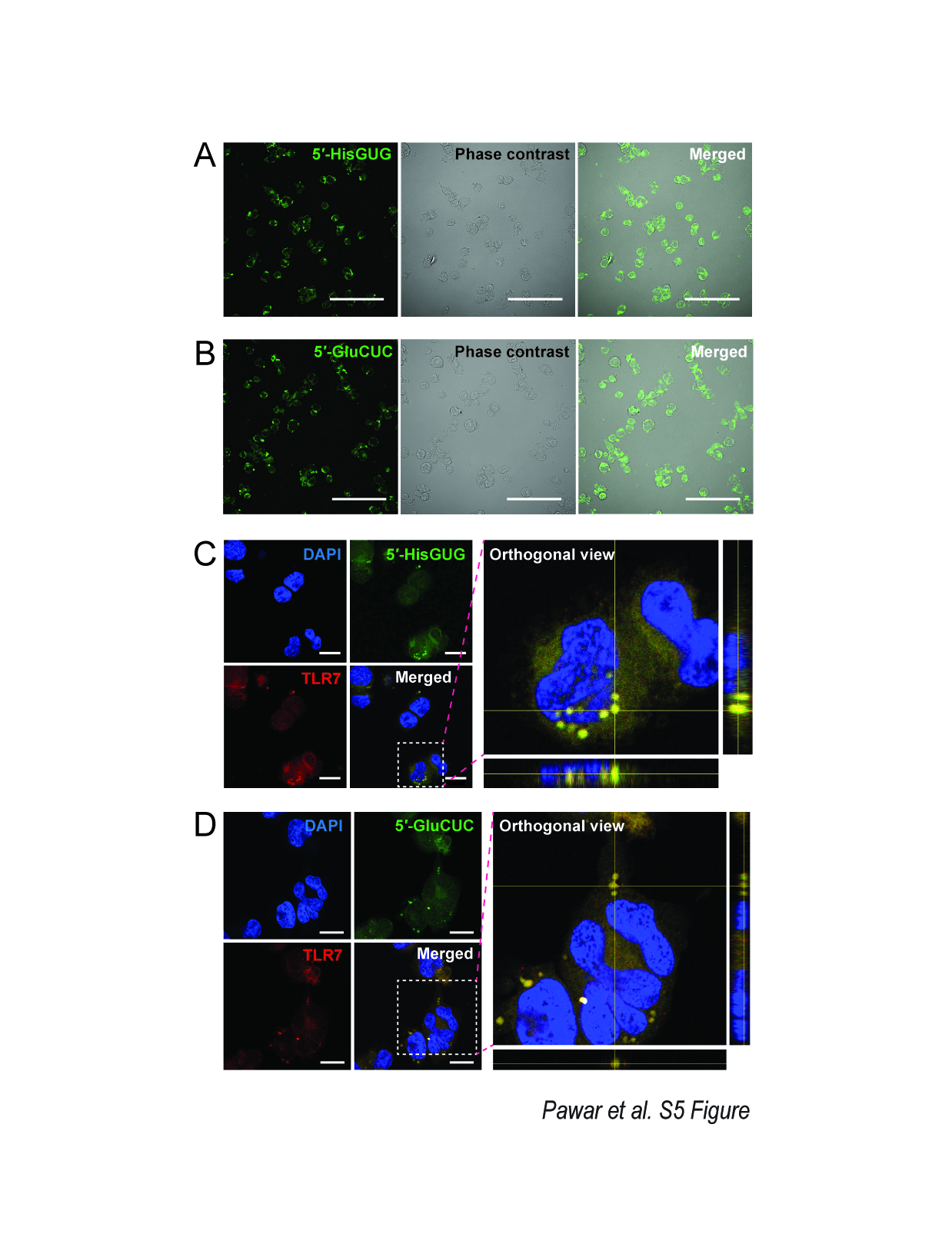

Supplement: S5 Fig — (A, B) Florescent end-labeled, synthetic 5′-tRNAHisGUG half (A) or 5′-tRNAGluCUC half (B) was transfected into HMDMs and observed in green. Scale bar, 20 μm. (C, D) EVs produced from host HMDMs containing the labeled 5′-tRNAHisGUG half or 5′-tRNAGluCUC half were isolated and applied to recipient HMDMs. Delivery of the labeled, EV-5′-tRNAHisGUG half (C) or EV-5′-tRNAGluCUC half (D) into endosomes was observed in green. Immunofluorescence staining of TLR7 is shown in red, and DNA was counterstained with DAPI in blue. Scale bar, 100 μm. Clear co-localization of the labeled 5′-tRNA halves and TLR7 was observed. EV, extracellular vehicle; HMDM, human monocyte-derived macrophage; TLR, Toll-like receptor; tRNA, transfer tRNA. (TIF) [file pbio.3000982.s005.tif]

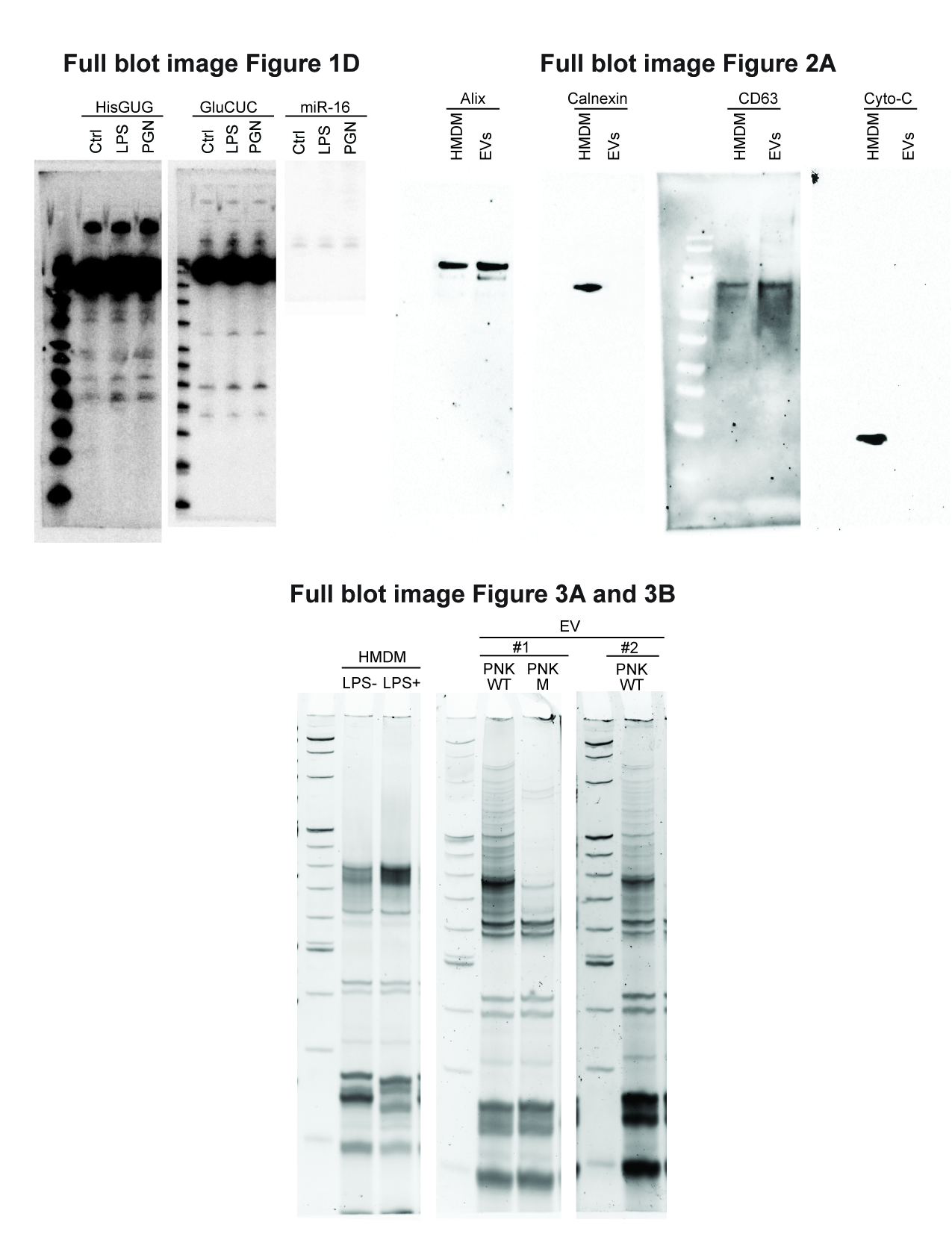

Supplement: S1 Raw Image — (TIF) [file pbio.3000982.s018.tif]

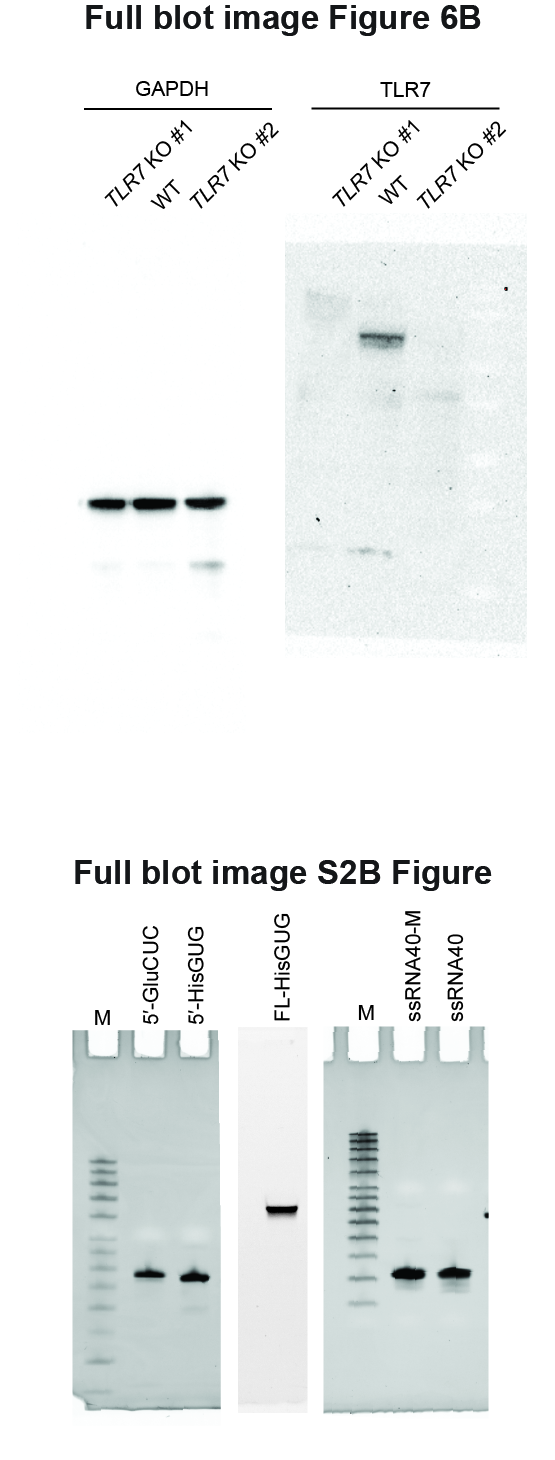

Supplement: S2 Raw Image — (TIF) [file pbio.3000982.s019.tif]
