# Supplementary material for: Infection-induced 5′-half molecules of tRNAHisGUG activate Toll-like receptor 7
Source: PLoS Biol. 2020 Dec 17;18(12):e3000982. doi: 10.1371/journal.pbio.3000982 (PMC7745994; doi:10.1371/journal.pbio.3000982)
Supplement: S1 Table — (PDF) [file pbio.3000982.s012.pdf]

S1 Table. Read numbers of sequence libraries

	Total	Quality filter	Mapping									
		3′AD-Trim/ 25-50 nt	tRNA	rRNA	mRNA	linc-RNA	mi-RNA	sno-RNA	misc-RNA	mito-RNA	unanno-tated	un-mapped
**HMDM**	39475445	37462099	1761814	28493497	1705115	337629	5285	440511	270275	830156	2726990	890827
**EV #1**	44513218	37567029	2473473	29572212	311698	216668	5193	147915	686353	60774	2647892	1444852
**EV #2**	35349311	29067447	2298613	21997754	326305	181717	8756	123601	495828	46149	2104178	1484546
