# Supplementary material for: Infection-induced 5′-half molecules of tRNAHisGUG activate Toll-like receptor 7
Source: PLoS Biol. 2020 Dec 17;18(12):e3000982. doi: 10.1371/journal.pbio.3000982 (PMC7745994; doi:10.1371/journal.pbio.3000982)
Supplement: S2 Table — RT-qPCR, quantitative reverse transcription PCR. (PDF) [file pbio.3000982.s013.pdf]

S2 Table. Sequences of TaqMan probes and primers for TaqMan RT-qPCR

Target	Probe/primer	Sequence (5′–3′)
5′-tRNA^{HisGUG} half	TaqMan	/5HEX/TAGTACTCT/ZEN/GCGTTGGAACACTGC GTTTGC/3IABkFQ/
	Forward	GCTCGCCGTGATCGTATAGT
	Reverse	GATCGTCGGACTGTAGAACTC
5′-tRNA^{GluCUC} half	TaqMan	/56FAM/CGCTCGAAC/ZEN/ACTGCGTTTG/3IAB kFQ/
	Forward	TCCCTGGTGGTCTAGTGG
	Reverse	GATCGTCGGACTGTAGAACTC

All synthetic probes and primes used in the present study were synthesized by Integrated DNA Technologies. TaqMan probes contain Hexachlorofluorescein (HEX), 6-carboxyfluorescein (FAM), and ZEN/Iowa Black as the fluorophore and quencher, respectively.
