# Supplementary material for: Infection-induced 5′-half molecules of tRNAHisGUG activate Toll-like receptor 7
Source: PLoS Biol. 2020 Dec 17;18(12):e3000982. doi: 10.1371/journal.pbio.3000982 (PMC7745994; doi:10.1371/journal.pbio.3000982)
Supplement: S3 Table — RT-qPCR, quantitative reverse transcription PCR. (PDF) [file pbio.3000982.s014.pdf]

S3 Table. Sequences of primers for stem-loop RT-qPCR

Target	Primer	Sequence (5′–3′)
miR-21	Stem-loop RT	GTCGTATCCAGTGCAGGGTCCGAGGTATTCGC ACTGGATACGACTCAACA
	Forward	CGGCGTAGCTTATCAGACT
	Reverse	GTGCAGGGTCCGAGGT
miR-150	Stem-loop RT	GTCGTATCCAGTGCAGGGTCCGAGGTATTCGC ACTGGATACGACCACTGG
	Forward	GACGTCTCCCAACCCTTG
	Reverse	GTGCAGGGTCCGAGGT
piR-3 (spike-in)	Stem-loop RT	GTCGTATCCAGTGCAGGGTCCGAGGTATTCGC ACTGGATACGACACCACT
	Forward	CGGCGTAGCTTATCAGACT
	Reverse	GTGCAGGGTCCGAGGT
