# Supplementary material for: Infection-induced 5′-half molecules of tRNAHisGUG activate Toll-like receptor 7
Source: PLoS Biol. 2020 Dec 17;18(12):e3000982. doi: 10.1371/journal.pbio.3000982 (PMC7745994; doi:10.1371/journal.pbio.3000982)
Supplement: S4 Table — RT-qPCR, quantitative reverse transcription PCR. (PDF) [file pbio.3000982.s015.pdf]

S4 Table. Sequences of primers for standard RT-qPCR

Target	Primer	Sequence (5′–3′)
MIP-1α	Forward	GCTGTCCTCCTCTGCACCAT
	Reverse	ATCTGCCGGGAGGTGTAGCT
MIP-1β	Forward	CATGCTAGTAGCTGCCTTCTGC
	Reverse	AGCTTCCTCGCGGTGTAAGA
TNFα	Forward	GAGCACTGAAAGCATGATCC
	Reverse	CGAGAAGATGATCTGACTGCC
ANG	Forward	AGAAGCGGGTGAGAAACAAAAC
	Reverse	AGTGCTGGGTCAGGAAGTGTG
IL-1β	Forward	CAGGCTGCTCTGGGATTCTC
	Reverse	CCTGGAAGGAGCACTTCATCT
IL-12p40	Forward	GAGTCTGCCCATTGAGGTCAT
	Reverse	AATTTTCATCCTGGATCAGAACC
TLR7	Forward	CCTTTCCCAGAGCATACAGC
	Reverse	GGACAGAACTCCCACAGAGC
TLR8	Forward	CAGAGCATCAACCAAAGCAA
	Reverse	GCTGCCGTAGCCTCAAATAC
RPLP0	Forward	CTATCATCAACGGGTACAAACGAG
	Reverse	CAGATGGATCAGCCAAGAAGG
GAPDH	Forward	GTCTTCACCACCATGGAGAAGG
	Reverse	ATGATCTTGAGGCTGTTGTCAT
U6 snRNA	Forward	TCGCTTCGGCAGCACATATAC
	Reverse	CGAATTTGCGTGTCATCCTTG
