# Supplementary material for: Infection-induced 5′-half molecules of tRNAHisGUG activate Toll-like receptor 7
Source: PLoS Biol. 2020 Dec 17;18(12):e3000982. doi: 10.1371/journal.pbio.3000982 (PMC7745994; doi:10.1371/journal.pbio.3000982)
Supplement: S5 Table — (PDF) [file pbio.3000982.s016.pdf]

S5 Table. Sequences of synthetic RNAs/DNAs

RNA	Sequence (5′–3′)
5′-HisGUG	GCCGUGAUCGUAUAGUGGUUAGUACUCUGCGUUG
5′-HisGUG-Mod	GCCGUGAUCGUAUAGDGGDDAGUACUCUGCGΨUG
5′-GluCUC	UCCCUGGUGGUCUAGUGGUUAGGAUUCGGCGCUC
FL-HisGUG	GCCGUGAUCGUAUAGUGGUUAGUACUCUGCGUUGUGGCCG CAGCAACCUCGGUUCGAAUCCGAGUCACGGCA
ssRNA40	GCCCGUCUGUUGUGUGACUC
ssRNA40-M	GCCCGACAGAAGAGAGACAC
miR-21	UAGCUUAUCAGACUGAUGUUGA
miR-150	UCUCCCAACCCUUGUACCAGUG
piR-3 (spike-in)	UGAGAGUGGCAUCUAAAUGUUUAGUGGU
AS-oligo	mG*mC*mC*mG*mT*mG*mA*mT*mC*mG*T*A*T*A*G*T*G*G* T*T*A*G*T*mA*mC*mT*mC*mT*mG*mC*mG*mT*mT*mG
Ctrl-oligo	mG*mT*mG*mT*mT*mC*mC*mG*mA*mT*G*T*G*G*C*T*C*T* G*G*A*C*T*mG*mG*mT*mA*mT*mC*mT*mA*mG*mT*mA

“D” designates dihydrouridine; “Ψ” designates peudouridine; “mN” designates 2′-*O*-methylated nucleotide; and “*” designates phosphorothioate bond.
