# Supplementary material for: Infection-induced 5′-half molecules of tRNAHisGUG activate Toll-like receptor 7
Source: PLoS Biol. 2020 Dec 17;18(12):e3000982. doi: 10.1371/journal.pbio.3000982 (PMC7745994; doi:10.1371/journal.pbio.3000982)
Supplement: S6 Table — (PDF) [file pbio.3000982.s017.pdf]

Table S6. Sequences of primers for the synthesis of dsDNA templates

Target	Primer	Sequence (5′–3′)
5′-tRNA^{HisGUG} half	Forward	GCTTAATACGACTCACTATAGCCGT
	Reverse	mCmAACGCAGAGTACTAACCACTATACGATCACGGC TACGATCACGGC TAGT
5′-tRNA^{GluCUC} half	Forward	CCTGCAGTAATACGACTCACTATAGGGAGAAGGGAC TGATGAGTCCGTGAGGACGAAACGGTACCCGGTACC GTCTCCCTGGTGGTCTAGTGGTTAG
	Reverse	mGmAGCGCCGAATCCTAACCACT
ssRNA40	Forward	GCTTAATACGACTCACTATAGCCCGT
	Reverse	mGmAGTCACACAACAGACGGGCTATAGT
ssRNA40-M	Forward	GCTTAATACGACTCACTATAGCCCGA
	Reverse	mGmUGTCTCTCTTCTGTCGGGCTATAGT

“mN” designates 2′-*O*-methylated nucleotide.
